# CyCoNP lncRNA establishes *cis* and *trans* RNA–RNA interactions to supervise neuron physiology

**DOI:** 10.1093/nar/gkae590

**Published:** 2024-07-11

**Authors:** Fabio Desideri, Alessandro Grazzi, Michela Lisi, Adriano Setti, Tiziana Santini, Alessio Colantoni, Gabriele Proietti, Andrea Carvelli, Gian Gaetano Tartaglia, Monica Ballarino, Irene Bozzoni

**Affiliations:** Center for Life Nano- & Neuro-Science of Istituto Italiano di Tecnologia (IIT), 00161 Rome, Italy; Center for Life Nano- & Neuro-Science of Istituto Italiano di Tecnologia (IIT), 00161 Rome, Italy; Department of Biology and Biotechnologies “Charles Darwin”, Sapienza University of Rome, 00185 Rome, Italy; Department of Biology and Biotechnologies “Charles Darwin”, Sapienza University of Rome, 00185 Rome, Italy; Department of Biology and Biotechnologies “Charles Darwin”, Sapienza University of Rome, 00185 Rome, Italy; Department of Biology and Biotechnologies “Charles Darwin”, Sapienza University of Rome, 00185 Rome, Italy; Center for Life Nano- & Neuro-Science of Istituto Italiano di Tecnologia (IIT), 00161 Rome, Italy; Department of Biology and Biotechnologies “Charles Darwin”, Sapienza University of Rome, 00185 Rome, Italy; Centre for Human Technologies (CHT), Istituto Italiano di Tecnologia (IIT), 16152 Genova, Italy; Department of Neuroscience, The Scripps Research institute, La Jolla, CA 92037, USA; Centre for Human Technologies (CHT), Istituto Italiano di Tecnologia (IIT), 16152 Genova, Italy; Department of Biology and Biotechnologies “Charles Darwin”, Sapienza University of Rome, 00185 Rome, Italy; Center for Life Nano- & Neuro-Science of Istituto Italiano di Tecnologia (IIT), 00161 Rome, Italy; Department of Biology and Biotechnologies “Charles Darwin”, Sapienza University of Rome, 00185 Rome, Italy

## Abstract

The combination of morphogenetic and transcription factors together with the synergic aid of noncoding RNAs and their cognate RNA binding proteins contribute to shape motor neurons (MN) identity. Here, we extend the noncoding perspective of human MN, by detailing the molecular and biological activity of CyCoNP (as Cytoplasmic Coordinator of Neural Progenitors) a highly expressed and MN-enriched human lncRNA. Through *in silico* prediction, *in vivo* RNA purification and loss of function experiments followed by RNA-sequencing, we found that CyCoNP sustains a specific neuron differentiation program, required for the physiology of both neuroblastoma cells and hiPSC-derived MN, which mainly involves miR-4492 and *NCAM1* mRNA. We propose a novel lncRNA-mediated ‘dual mode’ of action, in which CyCoNP acts *in trans* as a classical RNA sponge by sequestering miR-4492 from its pro-neuronal targets, including *NCAM1* mRNA, and at the same time it plays an additional role *in cis* by interacting with *NCAM1* mRNA and regulating the availability and localization of the miR-4492 in its proximity. These data highlight novel insights into the noncoding RNA-mediated control of human neuron physiology and point out the importance of lncRNA-mediated interactions for the spatial distribution of regulatory molecules.

## Graphical Abstract

**Graphical Abstract:**
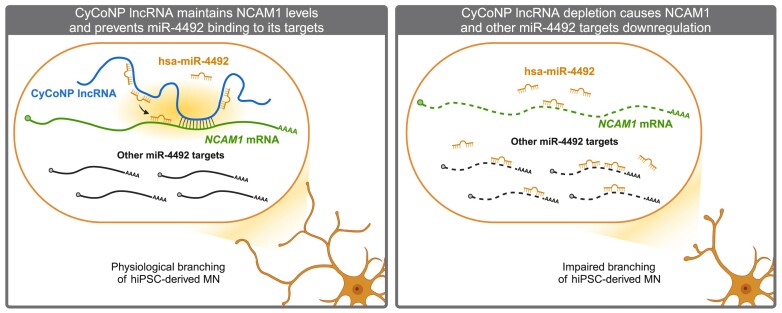

## Introduction

In mammals, motor neurons (MN) differentiation depends on the combined effect of several morphogenetic factors which define the spatial coordinates and direct the gene expression program of neural precursors to specific neural cell subtypes, in a gradient-driven fashion (1,2). In the last decade, with the development of high-throughput RNA sequencing, noncoding RNAs and especially long noncoding RNAs (lncRNAs), have emerged as key modulators of gene expression both at transcriptional and post-transcriptional levels in a cell type-specific pattern and in well-defined time frames for a variety of different tissues and organs, including the nervous system (3–9). Given their intrinsic nature, the biological function of lncRNAs is strongly influenced by their secondary structure and localization into specific subcellular compartments where they establish interactions with definite subsets of proteins and nucleic acids (10–12). Nuclear lncRNAs may participate as scaffolding molecules in the organization of subnuclear compartments which in turn influence many crucial steps of gene expression, such as topological organization of the chromatin and nucleation of ribonucleoprotein (RNP) condensates to control RNA transcription, splicing and export (13–16). Conversely, cytoplasmic lncRNAs have been described to modulate post-transcriptional steps of RNA metabolism, such as maturation, translation and stability by directly interacting with target mRNAs or by controlling the loading of specific RNA binding proteins (16–19). Finally, lncRNAs can act as miRNA sponges, targeting RNA expression by reducing miRNA availability in the cytoplasm (20,21). Although in the last decade many working models have been proposed for lncRNA mechanisms of action, a lot of work is still needed to experimentally validate the functional importance of this heterogeneous class of molecules (22). Here, we studied a lncRNA highly expressed in human neural and MN progenitors that we named CyCoNP, as Cytoplasmic Coordinator of Neural Progenitors. Particularly, we dissected CyCoNP mechanism of action as a strong regulator of NCAM1, a cell adhesion glycoprotein involved in neural development and differentiation, cell-to-cell adhesion, neurogenesis, neurite sprouting and cell migration (23–29). We showed that CyCoNP acts mainly in two ways: (i) *in trans*, as a classic sponge by sequestering miR-4492, which targets many neuronal mRNAs, including *NCAM1* and (ii) *in cis*, by directly interacting with *NCAM1* mRNA to control the miRNA abundance in its proximity and thus facilitating the loading of the miRNA on the *NCAM1* mRNA. Finally, the depletion of CyCoNP caused alterations in cellular physiology, primarily concerning cell motility in SK-N-BE cells and branching activity in hiPSC-derived MN. Our results highlight the importance of RNA–RNA interactions and point out CyCoNP lncRNA as a pivotal regulator of NCAM1 expression and its related pathways in the context of neurogenesis.

## Materials and methods

### Cell culture

SK-N-BE cells were cultured in growth medium with RPMI-1640 (Sigma-Aldrich, cat. no. R0883), 10% fetal bovine serum (FBS; Sigma-Aldrich), 1% GlutaMAX (Thermo Fisher Scientific, cat. no. 35 050 061), 1% Pen/Strept (Thermo Fisher Scientific, cat. no. 15 070 063), 1% Sodium Piruvate (Thermo Fisher Scientific, cat. no. 11 360 070). Differentiation was induced by adding differentiation medium: RPMI-1640 (Sigma-Aldrich), 2.5% heat-inactivated FBS (Sigma-Aldrich), 1% GlutaMAX (Thermo Fisher Scientific), 1% Pen/Strept (Thermo Fisher Scientific), 1% sodium piruvate (Thermo Fisher Scientific) and 10 μM retinoic acid (RA; Sigma-Aldrich, cat. no. R2625). N2a cells were cultured in growth medium with DMEM High glucose (Sigma-Aldrich, cat. no. D6429), 10% fetal bovine serum (FBS; Sigma-Aldrich), 2 mM l-glutamine (Sigma-Aldrich, cat. no. G7513) and 1% Pen/Strept (Thermo Fisher Scientific, cat. no. 15 070 063). hiPSCs were derived, maintained and induced to differentiate towards MN fate following the methods described in (30).

### RNA preparation and analysis

Total RNA from cells was extracted with the Direct-zol RNA Purification Kit (Zymo Research) and reverse transcribed with PrimeScript RT reagent Kit (Takara-Clonetech), SuperScript VILO cDNA Synthesis Kit (Thermo Fisher Scientific) or miRCURY LNA RT Kit (Qiagen). For mRNAs, RT-qPCR analysis was performed with SYBR Green Power-UP (Life Technologies), using the housekeeping GAPDH (glyceraldehyde-3-phosphate dehydrogenase) or ATP5O (ATP synthase peripheral stalk subunit OSCP) genes as an internal controls. For miRNAs, RT-qPCR analysis was performed with SYBR Green PCR Master Mix (Qiagen) for miRNAs. Each reaction was performed in three technical replicates and according to manufacturer's protocol.

### Protein analysis

For Western Blot analysis, proteins were collected in RIPA Protein buffer completed with Proteinase Inhibitor Complex (PIC) 100X, loaded on 4–12% bis–tris–acrylamide gel (Thermo Fisher Scientific), and transferred to a nitrocellulose membrane (Millipore). The membrane was blocked in 10% milk and hybridized with the specific antibodies overnight at 4°C at the appropriate dilutions, according to manufacturers’ instructions. After three washes in TBST, the filter was hybridized with the corresponding secondary antibody for one hour at room temperature. Protein detection was carried out with the Long-Lasting Chemiluminescent Substrate (EuroClone) using ChemiDoc MP System. Images were analyzed using Image Lab Software (BioRad). See Supplementary Table S1 for antibodies details.

### Plasmid construction

For the Luciferase constructs, the full-length CyCoNP cDNA and the *NCAM1* 3′ UTR entire sequence (WT) were PCR-amplified from SK-N-BE cells using Clone AMP PCR HIFI (Takara-Clontech). PCR fragments were cloned downstream of the Renilla luciferase stop codon in the psiCHECK-2 plasmid (Promega) previously linearized with NotI (NEB) and XhoI (NEB) enzymatic digestion by using T4 DNA ligase (Thermo Fisher Scientific). The plasmid lacking miR-4492 binding sites on CyCoNP sequence (CyCoNP ΔmiR-4492 plasmid) was obtained from the CyCoNP WT luciferase plasmid by performing a deletion of the sequence including the first and second sites, together with a deletion of the third site. In order to achieve this, the plasmid was linearized by inverse PCR and all the sequence spanning the first and third site was removed. The sequence between the second and third sites was then reinserted through In-Fusion cloning (Takara-Clonetech).

The luciferase plasmid lacking miR-4492 binding site on *NCAM1* 3′UTR sequence was obtained by inverse PCR on the *NCAM1* 3′UTR WT construct.

To obtain the CyCoNP WT and *NCAM1* coding DNA sequence (CDS) overexpressing plasmids, the entire CyCoNP sequence and the *NCAM1* CDS were PCR-amplified from cDNA of SK-N-BE cells using Clone AMP PCR HIFI (Takara-Clontech). These sequences were cloned with In-Fusion cloning (Takara-Clonetech) downstream of the CMV promoter contained in pcDNA 3.1 ^(+)^ plasmid (Thermo Fisher Scientific), previously linearized with HindIII (NEB) and NotI (NEB) enzymatic digestion. To clone the mutant version of CyCoNP (CyCoNP mutant), lacking the region targeted by si-CyCoNP, the cloning strategy was the same, except that a sequence of CyCoNP lacking 150 nt at its 3′ end was amplified. All the oligonucleotides employed to generate these constructs are listed in Supplementary Table S1.

### CRISPR/Cas9 genome editing for CyCoNP KO hiPSC clones generation

sgRNAs were designed using Zhang design tools at http://crispr.mit.edu/, ordered as single-strand DNA oligos and cloned in the PX330 vector (Addgene) encoding wild type (WT) Cas9 protein. HR110PA-1 (System Biosciences) was used as a backbone to create the donor vector (DONOR). Poly(A)/2 × MAZ sequence (PAS; 31) was cloned into the donor vector followed by a Neomycin resistance cassette using In-Fusion® HD Cloning Plus Kit (Cat. #638 910). Homology arms (HA) were designed to be longer than 500  nt and amplified by PCR on hiPSCs gDNA (Clone AMP PCR HIFI, Takara-Clontech) and cloned in MCS1 (upstream of PAS) and MSC2 for the left and right arms, respectively. hiPSCs were transfected on matrigel-coated dishes through the Neon Transfection System (Life Technologies), using 100 μl tips in R buffer with the following settings: 1200 V, 30 ms, 1 pulse. Selection was carried out in 800 μg/ml G418 for 5 days. Single clones were amplified and genotyped.

### Cell transfection and dual-luciferase reporter assay

SK-N-BE cells were plated (100 000 cells/well of a 12-well plate) in growth medium (FBS 10%, GlutaMAX 1%, Pen/Strept 1%, sodium pyruvate 1% and RPMI-1640) and transfected 24 hours later with 60 nM of siRNA pool targeting CyCoNP (si-CyCoNP) or 75 nM of siRNA pool targeting *NCAM1* mRNA (si-NCAM1) or 100 nM of LNA-CyCoNP or 100 nM of LNA targeting miR-4492 or the respective scrambled controls (si-SCR or LNA-SCR) using Lipofectamine RNAiMAX Transfection Reagent (Thermo Fisher Scientific) according to the manufacturer's specifications. 18 hours after transfections cells were exposed to differentiation medium (FBS USA 2,5%, GlutaMAX 1%, Pen/Strept 1%, sodium pyruvate 1%, RA 10 μM and RPMI-1640) for additional 36 hours prior to cell collection. For the CyCoNP luciferase assays: cells were co-transfected with 100 ng of psiCHECK-2 Luciferase plasmid containing the CyCoNP sequence and with LNA molecules targeting either miR-4492 or miR-1249–5p at a concentration of 60 nM (see Supplementary Table S1). For the CyCoNP ΔmiR-4492 luciferase assays: cells were co-transfected with 100 ng of psiCHECK-2 Luciferase plasmid containing the CyCoNP ΔmiR-4492 sequence and with LNA molecules targeting either miR-4492 or a scramble control at a concentration of 60 nM. For the *NCAM1* 3′ UTR luciferase assays: cells were co-transfected with 100 ng of psiCHECK-2 Luciferase plasmid containing *NCAM1* 3′ UTR WT sequence or its mutant version, in combination with: 60 nM of siRNA pool targeting CyCoNP, or 60 nM of LNA targeting miR-4492, or 100 ng pcDNA 3.1 ^(+)^ plasmid (Thermo Fisher Scientific) containing the CyCoNP sequence, or 50 nM of LNA-CyCoNP. All the experiments were performed including a scramble or negative control, specific for each experimental condition. The rescue experiment was performed by co-transfecting cells with CyCoNP siRNAs together with pcDNA 3.1 ^(+)^ containing a mutated form of CyCoNP. All transfections were performed using Lipofectamine 2000 (Thermo Fisher Scientific) according to the manufacturer's specifications.

N2a cells were plated (250 000 cells/well of a 12-well plate) in growth medium (FBS 10%, l-glutamine 2 mM, Pen/Strept 1% and DMEM High glucose) and transfected 24 hours later with 100 ng of CyCoNP luciferase plasmid or *NCAM1* 3′ UTR WT plasmid, together with 12.5 or 25 nM of miR-4492 mimics or mimic negative control. The transfections were performed using Lipofectamine 2000 (Thermo Fisher Scientific) according to the manufacturer's specifications. Luciferase activity was measured in GloMax-Multi^+^ Detection System (Promega), using the Dual-Luciferase Reporter Assay System (Promega).

### Scratch-wound assay

SK-N-BE cells were plated on 12-well dishes (100 000 cells/well) in growth medium for 24 h and transfected with si-SCR or si-CyCoNP, as described in the previous section; or with 60 nM of LNA-SCR or LNA targeting miR-4492; or with 100 ng of an overexpression construct encoding for the *NCAM1* CDS or an empty vector used as a control. Cells were scratched with 1000 μl tips 18 h after transfections and exposed to starvation medium (FBS 0%) with RA 10 μM. 24 h later, images were taken, and cell number quantification was performed using ImageJ software.

### Cellular fractionation

SK-N-BE cells were subject to subcellular fractionation using the Ambion PARIS Kit (AM1921, Life Technologies). After RNA extraction, equal volumes of cytoplasmic and nuclear RNA were retro-transcribed and analyzed by RT-qPCR. Normalizations were based on the total amount of RNA.

### Immunofluorescence and neuron morphology assessment

hiPSC-derived MN were cultured on precoated glass coverslips (0.01% poly‐l‐ornithine/Murine Laminin 20 μg/ml, Sigma) and then fixed in 4% paraformaldehyde (Electron Microscopy Sciences, Hatfield, PA) for 20 min at room temperature, washed with PBS and then permeabilized and blocked with 0.1% Triton X‐100/3% BSA for 30 min at room temperature. Subsequently, cells were incubated with primary antibodies (anti‐Islet 1/2, anti-ChAT, anti-MAP2) in 0.1% Triton X‐100/2% BSA overnight at 4°C. After washing with PBS, cells were labelled with secondary antibodies (Goat anti-Mouse-Cy3, Donkey anti-chicken 488, Donkey anti-goat 555) in BSA 2%/PBS for 45 min at room temperature. Nuclei were counterstained with DAPI solution (1 μg/ml in PBS) for 5 min at room temperature, and the coverslips were mounted using ProLong Diamond Antifade Mountant (Thermo Fisher Scientific, P‐36 961). Cells were imaged using an inverted confocal Olympus IX73 microscope equipped with a Crestoptics X‐LIGHT V3 spinning disk system and a Prime BSI Express Scientific CMOS camera. The images were acquired as 16‐bit 2048 × 2048 pixel files by using a LUCPlanFLN 20X objective (NA 0.45) and a UPLANSApo 60X (NA 1.35) oil objective, and were collected with the MetaMorph software (Molecular Devices). Single-cell morphology characterization was performed on Z-stack confocal images by using NeuronJ ImageJ plugin (51,52). Tracing data were collected to evaluate dendrite elongation (#sum length of all neurites per cell; #sum length of primary, #secondary, #tertiary and quaternary dendrites per cell) and dendrite branching quantification (#total number of dendrites per cell; #number of primary, #secondary, #tertiary and quaternary dendrites per cell).

### Cross-linking and immunoprecipitation (CLIP) assay

Plated SK-N-BE cells (D 1.5) were UV cross-linked at 254 nm with 4 000 μJoules/cm² energy using a Stratalinker and harvested in NP40 lysis buffer pH 7.5 (50 mM Hepes–KOH; 150 mM KCl; 2 mM EDTA; 1 mM NaF; 0.5% NP40; 0.5 mM DTT; 1 × PIC) and incubated on ice for 10–15 min followed by centrifugation at 18 000 x g for 10 min at 4°C. Resulting cellular lysates were incubated (overnight on a rotating wheel, at 4°C) with 30 μl of Dynabeads Protein G magnetic particles (Invitrogen) preincubated with either 8 μg of AGO2 Antibody (MA5-23515, Invitrogen) or mouse IgG (sc-2025, Santa Cruz, see Supplementary Table S1). After incubation, beads were washed with a High-Salt buffer (50 mM Hepes-KOH; 500 mM KCl; 0.5 mM DTT; 0.05% NP40). Before RNA extraction, 1/4 of the cell lysate was heated for 5 min at 95°C, and the supernatant was collected and resuspended in Protein elution buffer (4 × Laemmli sample buffer [BioRad]) with DTT 50 mM and analyzed by western blot. RNA fraction was treated with Proteinase K (AM2546, Thermo Fisher Scientific) for 30 min at 50°C; the samples were then placed for 10 min at 95°C, and finally, the RNA was extracted using Direct-zol RNA Purification Kit (Zymo Research) with on-column DNase treatment, according to the manufacturer's instructions.

### RNA pull-down assay

Native RNA pull-down on total extract from SK-N-BE cells was performed according to (32). Briefly cells were harvested in lysis buffer (Tris–HCl pH 7.5 50 mM, NaCl 150 mM, MgCl_2_ 3 mM, NP40 0.5%, EDTA 2 mM, DTT 1 mM; 1 × PIC, and RNase inhibitors) and incubated on ice for 10–15 min, prior to centrifugation at 15 000 × g for 15 min. After lysis and clearing by centrifugation, 1 mg of extract was diluted in a 1:2 ratio with hybridization buffer containing Tris–HCl pH 7.5 100 mM, NaCl 300 mM, MgCl_2_ 1 mM, SDS 0.2%, formamide 15%, NP40 0.5%, EDTA 10 mM, DTT 1 mM, 1 × PIC and RNase inhibitors. 10% of the total extract was collected for Input (INP). 100 pmol of previously heat-denatured biotinylated probes were added (see Supplementary Table S1). To enhance RNA recovery 2.5% dextran sulfate was then added to the PD and control samples (LacZ). After 4 h of incubation at 4°C, 0.1 ml of streptavidin magnasphere paramagnetic beads (Promega) were added to pull down the complex, and the mixture was incubated for 1 h at room temperature. Beads were then washed 4 times with hybridization buffer and RNA was extracted and DNase treated for further analyses (see RNA-Seq section). Pull-down (PD) RT-qPCR results were represented as a percentage of PD/input signal (% of input). Psoralen (AMT)-crosslinked RNA pull-down assay was performed as described in (33), with some modifications in the first steps. Briefly, 10 × 10^6^ SK cells for each biological replicate were pelleted, resuspended in 1 ml of ice-cold PBS with Ca^2+^/Mg^2+^ supplemented with 0.5 mg/ml of 4′-aminomethyl-4,5′,8-trimethylpsoralen (AMT, Sigma-Aldrich), and cross-linked at 365 nm for five 2-min cycles. 1 volume of Guanidinium Hydrochloride 6 M was added to 1 volume of AMT. The lysate was subdivided into 250 μl aliquots. To each aliquot, 25 μl of a 20 mg/ml solution of Proteinase K (Ambion) and 6.5 μl of 20% SDS were added. The samples were then incubated at 65°C for 1 h. RNA was isolated through phenol-chloroform precipitation and extracted using the Qiagen RNA extraction kit. The recovered RNA fraction was used for the RNA pull-down procedure.

### RNA-sequencing samples preparation and analysis

Illumina Stranded Total RNA Prep Ligation with Ribo-Zero Plus was used to prepare cDNA libraries for RNA‐Seq. The sequencing reactions, performed on an Illumina Novaseq 6000 Sequencing system at IIT-Istituto Italiano di Tecnologia (Genova, Italy), produced an average of 99,5 million 100 nucleotide long paired‐end reads per sample. Trimmomatic v0.39 (34) with parameters: *-PE ILLUMINACLIP:adapter_path:2:30:10:8:3:true LEADING:3 TRAILING:3 SLIDINGWINDOW:4:20* and Cutadapt v3.2 (35) with parameters: *-u 1 -U 1 –nextseq-trim = 20 –trim-n* were used to remove adapter sequences and poor quality bases; minimum read length after trimming was set to 18. Reads aligning to rRNAs were filtered out; this first alignment was performed using Bowtie2 software v2.4.2 (36). STAR software v2.7.7a (37) was used to align reads to GRCh38 genome using the following parameters: *–outSAMstrandField intronMotif –outSAMattrIHstart 0 –outFilterType BySJout –outFilterMultimapNmax 20 –alignSJoverhangMin 8 –alignSJDBoverhangMin 1 –outFilterMismatchNmax 999 –outFilterMismatchNoverLmax 0.04 –alignIntronMin 20 –alignIntronMax 1 000 000 –alignMatesGapMax 1 000 000 –outFilterIntronMotifs RemoveNoncanonical –peOverlapNbasesMin 10*. Uniquely mapping fragments were counted for each annotated gene (ensemble release 99) using –quantMode GeneCounts parameter of STAR aligner. DESeq2 R package v 1.32.0 (38) was used to compare si-SCR and si-CyCoNP conditions. RNAs with average FPKM expression levels <1 in both analyzed conditions were filtered out. RNAs with log_2_FC > 1 and adjusted *P*-value <0.05 were defined ‘upregulated’ in si-CyCoNP condition while those with log_2_FC <–1 and adjusted *P*-value <0.05 were defined ‘downregulated’. All the others were labelled as ‘invariant’. Gene ontology was performed using WebGestalt R tool (v0.4.4; 39).

Isoform quantification in SK-N-BE cells and in hiPSC-derived MN was performed using Salmon software (v1.6.0; 40) on transcriptome sequences retrieved from Ensembl (release 99; 41) using biomaRt (https://bioconductor.org/packages/release/bioc/html/biomaRt.html) (v2.48.3). Raw RNA-Seq data from hiPSC- derived MN were analyzed from GSE94888 (42) with the following pre-processing procedure: Cutadapt (v3.2; 35) and Trimmomatic (v0.39; 34) were used in order to remove adapter sequences and low quality bases; the minimum read length after trimming was set to 35.

Libraries for nanopore sequencing were prepared from total RNA according to protocols provided by Oxford Nanopore (Oxford Nanopore Technologies) for PCR-cDNA barcoding (SQK-PCB109). For reverse transcription, Maxima H Minus Reverse Transcriptase (Thermo Fisher Scientific) was used for all samples. A total of 100 ng was retrotranscribed for input (4 ml) and 9 ml for PD and LacZ samples. Two reactions of PCR were pulled together for input samples and four reactions for the remaining samples. 15 PCR cycles were run for all samples. The sequencing experiment was performed on the FLO-MIN106 flowcell using Oxford Nanopore MK1c with the following configuration: MinKNOW 22.12.15, Bream 7.4.8, Configuration 5.4.7 and MinKNOW Core 5.4.3 (available at https://community.nanoporetech.com). Following the sequencing run, basecalling was performed on the FAST5 data using Guppy v. 6.4.6 (available at https://community.nanoporetech.com) with the following parameters: *–enable_trim_barcodes, –trim_adapters, –trim_primers, –recursive, –min_qscore 9, –compress_fastq, -x ‘cuda:0’, –num_callers 14, –gpu_runners_per_device 8, –chunks_per_runner 768, –chunk_size 500*; only the reads flagged as ‘pass’ (average quality higher than or equal to 9) were kept for downstream analyses. The quality of the reads was assessed using PycoQC v2.5.2 (43). Nanofilt v2.8.0 (44) was used to remove reads shorter than 100 nucleotides. The resulting reads were then aligned to the *Homo sapiens* GRCh38 reference genome (45) using Minimap2 v2.24-r1122 (46) with the parameter *-ax splice*, which allows spliced alignments. Transcript quantification was performed using Bambu v1.1.2 without the discovery of new isoforms (*discovery = FALSE*) (47), utilizing the Ensembl 99 annotation (41) as a reference. Gene-level counts were then employed for differential abundance analysis using DESeq2 v1.30.0 (38), following the exclusion of genes with a total count of supporting reads less than 10. CyCoNP binders were identified as those significantly enriched (log_2_ fold-change > 1 and adjusted *P*-value < 0.05) in the pull-down versus Input comparison, but not in the LacZ versus Input comparison. In order to obtain more stringent results, log_2_ fold-change threshold in the LacZ versus Input comparison was imposed at > 0.

### *In silico* prediction of miRNA targeting sites and RNA–RNA interactions

miRNA binding sites prediction on CyCoNP sequence were performed using miRanda software (v3.3a; 48) setting minimum prediction score to 140 and energy threshold at –15 kcal/mol.

For miRNA-target prediction within si-CyCoNP down-regulated mRNAs three softwares were implemented: miRanda prediction with the described parameters; PITA (49) standard parameters were applied filtering out results with ΔG energy major than 0 and with pairing energy major than –15 kcal/mol; finally TargetScanV8 (50) predictions were retrieved from (https://www.targetscan.org/cgi-bin/targetscan/data_download.vert80.cgi) selecting both conserved and non-conserved for all predicted interactions. Moreover, in order to reduce sequence redundancy, for each gene the protein-coding isoform most expressed in SK-N-BE cells in si-SCR condition was selected as representative. Annotated 3′UTR regions (Ensemble 99) were used to assess miRNA binding to mRNAs.

### Statistical analysis

Data are expressed as mean values, and error bars represent SD or SEM. Statistical differences were analyzed by a two-tailed unpaired or paired Student's *t*-test as specified for each experiment. A *P*-value <0.05 was considered as statistically significant.

## Results

### CyCoNP is a functional lncRNA highly enriched in neural progenitors

**Figure 1.**
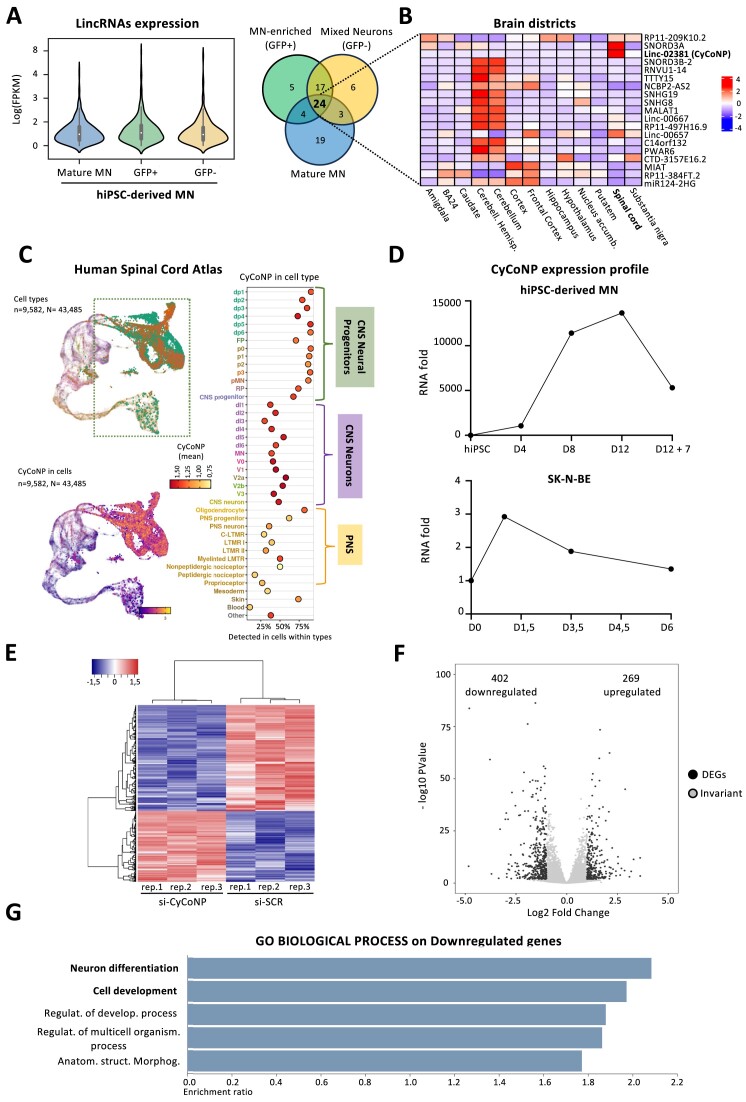
CyCoNP lncRNA expression profile and its transcriptome regulation in neuronal cells. (**A**) Left panel: violin plot showing the log(FPKM) of expressed lincRNAs (FPKM > 1) in hiPSC-derived MN from a mixed population (MN mix), MN-enriched (GFP+) and MN-depleted (GFP–) cells. For each condition the average FPKM of samples was taken into consideration. Right panel: Venn diagram displaying the intersection of top expressed lincRNAs in MN mix, GFP + and GFP– cells. FPKM: Fragments Per Kilobase of transcript per Million mapped reads. (**B**) Heatmap showing the enrichment of the selected top commonly expressed lincRNAs in MN mix, GFP+ and GFP– cells among the different brain districts as retrieved from GTEx portal (GTEx analysis 2017-06-05_v8_RNASeQCv1.1.9). Only lincRNAs with available gene ID on GTEx portal are shown. (**C**) UMAP plot showing CyCoNP expression in single-cell transcriptomes of human embryonal neural tube. Colored braces demarcate cellular subtypes corresponding to the main different populations. Neural progenitors are demarcated in green (see https://journals.biologists.com/dev/article/148/15/dev199711/271192/Single-cell-transcriptome-profiling-of-the-human for details). CNS: Central Nervous System; PNS: Peripheral Nervous System. (**D**) RT-qPCR analysis of CyCoNP along hiPSC-derived MN differentiation (upper panel) and SK-N-BE cell line (lower panel) differentiation. Data are normalized over *ATP5O* (for hiPSC-MN) or *GAPDH* (for SK-N-BE) transcripts. *n* = 1 biological replicate. (**E**) Heatmap visualization from RNA-seq analysis of SK-N-BE cells at 1.5 days of differentiation (D 1.5) treated with si-SCR or si-CyCoNP. Plot was produced by heatmap3 (https://cran.r-project.org/web/packages/heatmap3/vignettes/vignette.pdf). Expression values were calculated as FPKM, were log_2_-transformed and mean-centered. FPKM: Fragments Per Kilobase of transcript per Million mapped reads. (**F**) Volcano plots showing differential gene expression from transcriptome analysis of SK-N-BE cells (D 1.5) treated with si-SCR or si-CyCoNP. The numbers of the relative down and upregulated genes in the two conditions are shown. DEGs: Differentially Expressed Genes. (**G**) Gene Ontology (GO) enrichment analysis performed by WEBGESTALT (http://www.webgestalt.org) on down-regulated genes in SK-N-BE cells (D 1.5) treated with si-SCR or si-CyCoNP. Bars indicate the top categories of Biological processes in decreasing order of enrichment ratio. All the represented categories show a False Discovery Rate (FDR) value <0.05.

With the aim to isolate functional lncRNAs active along MN differentiation, we took advantage of a transcriptome profiling performed in a MN model system derived from human induced pluripotent stem cells (hiPSC; 42). We narrowed our analysis by selecting the most abundant long intergenic noncoding RNAs (lincRNAs) among different subcellular populations (Figure 1A). We selected 24 common highly expressed lincRNAs (Figure 1A; Supplementary Figure S1A) and we found that, among the brain districts available in GTEx portal database (GTEx analysis 2017-06-05_v8_RNASeQCv1.1.9), linc-02381 and SNORD3A displayed the highest expression levels in the spinal cord (Figure 1B). However, among the other tissues, linc-02381 was also enriched in the Tibial Nerve, that comprises peripheral MN components (Supplementary Figure S1B). Finally, conservation analysis using the TransMap V5 algorithm (https://genome.ucsc.edu/cgi-bin/hgTrackUi?db=hg38&g=transMapV5) identified putative linc-02381 homologous transcripts in 11 mammalian species (Supplementary Table S3), making it an intriguing candidate for further studies. On this basis, we turned to the analysis of the linc-02381 genomic locus (ENSG00000250742) from which, according to Ensembl 110 (53) different RNA isoforms are produced but only one (Linc-02381-201, here renamed CyCoNP (Cytoplasmic Coordinator of Neural Progenitors), was well detectable from RNA-seq data of hiPSC-derived MN (Supplementary Figure S1C, D). To go deeper at single cell resolution, we interrogated the single cell transcriptome atlas of the human developing spinal cord (54) finding that, between all the different cellular populations, Neural Progenitors (NP) display the greatest expression levels of CyCoNP (Figure 1C). We then started to profile CyCoNP expression in two different cellular model systems. By differentiating hiPSCs toward the MN fate (30; Supplementary Figure S1E) we observed that CyCoNP expression is highly induced during the differentiation process peaking between day 8 and 12 (Figure 1D, upper panel), that coincide with the neural and MN progenitors state (42), and decreases along with MN maturation (Supplementary Figure S1E; Figure 1D, upper panel). A comparable output was found when we profiled CyCoNP expression during the differentiation process of SK-N-BE cells, a cell type commonly used to recapitulate neurogenesis (55). Again, we observed a peak of CyCoNP expression at the beginning of differentiation (around day one) with a decrease in the following days of cell maturation (Figure 1D, lower panel). Given the high heterogeneity of hiPSC-derived MN and their reluctance to cell transfection (42), we proceeded with the functional analysis of CyCoNP in SK-N-BE cell line. As a first step into the characterization of this lncRNA we performed loss of function experiments at day 1.5 of differentiation followed by RNA-Sequencing, using a pool of four siRNAs targeting different portions of the second exon of CyCoNP (Figure 1E; Supplementary Figure S1C). Sequence analysis confirmed the strong efficacy of siRNAs in abolishing CyCoNP expression (Supplementary Figure S1F, left panel) and led to the identification of 671 differentially expressed genes (DEGs; false discovery rate [FDR] <0.05, si-SCR versus si-CyCoNP, Supplementary Table S2), 402 of which were downregulated and 269 were upregulated (Figure 1F; Supplementary Table S2). A Gene Ontology (GO) term enrichment analysis performed separately on the DEGs revealed that CyCoNP-downregulated genes mainly cluster into ‘Neuron differentiation’ and ‘Cell development’ categories (Figure 1G, Supplementary Table S2). Interestingly, among the most affected transcripts we found genes with relevant roles in nervous system development, neurite outgrowth, neurotransmission signaling, synapses formation and maturation such as NCAM1, SLC18A3, RPH3A and CHRM1 (24,56–58); Supplementary Figure S1F, right panel). When the same analysis was performed on the upregulated genes, we found a poor enrichment into specific ontology terms with only ‘extracellular structure organization’ category displaying a significant enrichment (Supplementary Figure S1G, Supplementary Table S2). Taken together, these data suggest a role of CyCoNP in a specific window of neurogenesis, which opens the door to a deeper molecular and functional characterization.

### CyCoNP can bind microRNAs in neuronal cells to control gene expression

Over the years, plenty of diverse lncRNAs mechanisms of action have been proposed, contributing to detail the involvement of these molecules in almost every aspect of cells physiology (16,8). A first step into the inspection of lncRNAs function is the study of its subcellular localization (11,59). On this basis, we assessed CyCoNP cellular localization in our model system by performing biochemical subcellular fractionation in SK-N-BE cells. RT-qPCR analysis shows that CyCoNP is highly enriched in the cytoplasm at levels comparable to *GAPDH* mRNA, used as a control, suggesting a potential role of the lncRNA in this cellular compartment (Supplementary Figure S2A). Multiple data have shown that cytoplasmic lncRNAs may bear micro ORFs which can be translated and have a biological function (60,61). To assess the coding potential of CyCoNP, two independent softwares, CPC2 and CPAT, were employed. Both programs categorized CyCoNP as a noncoding RNA. In detail, to acquire a comprehensive evaluation, CyCoNP coding potential metrics including Fickett score, hexamer composition, predicted peptide length, and isoelectric point (Supplementary Figure S2B–E) were compared to a control set comprised of 50 protein-coding and 50 long noncoding RNAs (according to Ensembl release 99, 41). Importantly, this control set was chosen to have similar lengths to CyCoNP (within +/–10% of its length) and exhibits no significant length variations between mRNAs and lncRNAs (Supplementary Figure S2F). This analysis revealed that CyCoNP scores for all evaluated metrics resemble more closely to those of lncRNAs compared to mRNAs, thus excluding the presence of a micro ORF.

**Figure 2.**
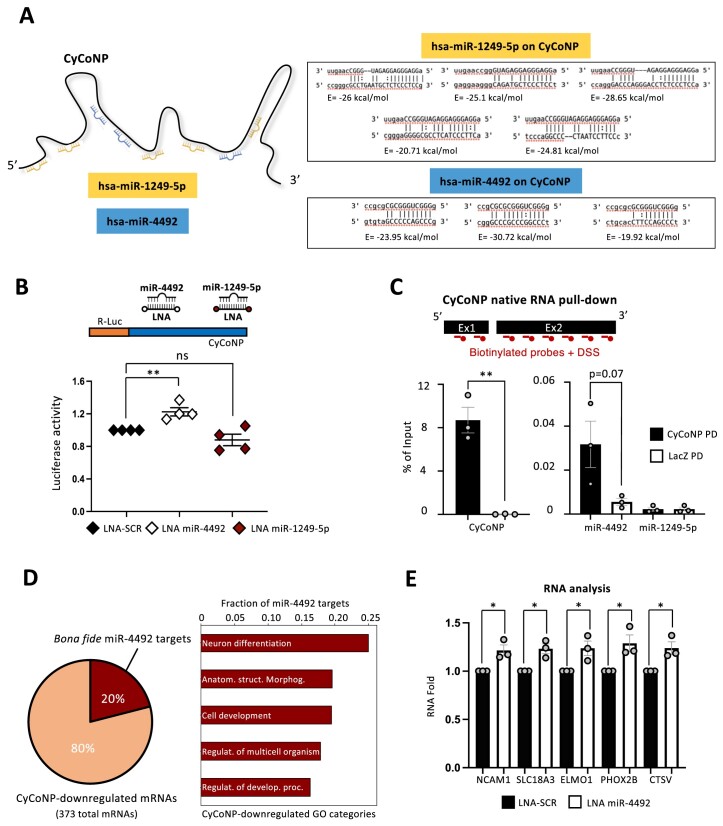
CyCoNP can bind miRNAs in neuronal cells. (**A**) Left: schematic representation of CyCoNP sequence and the relative position of the binding sites of the top two miRNAs predicted to interact with the lncRNA. Right: visualization of each predicted binding site of miR-1249-5p and miR-4492 on CyCoNP sequence. For each site, the energy (*E*) of interaction retrieved by miRanda is shown. (**B**) Upper panel: schematic representation of the CyCoNP luciferase-based reporter construct. The full-length sequence of CyCoNP lncRNA was cloned downstream of the Renilla luciferase ORF (orange). As indicated, the construct was co-transfected in SK-N-BE cells with a control LNA (LNA-SCR) or LNA targeting miR-4492 and miR-1249-5p. **See Materials and methods for details**. Lower panel: quantification of Renilla luciferase activity in SK-N-BE cells co-transfected with the CyCoNP luciferase construct and LNA-SCR or LNA targeting miR-4492 and miR-1249-5p. Data represent the mean luciferase activities ± SEM of four biological replicates. (**C**) Upper panel: schematic representation of CyCoNP RNA pull-down assay in SK-N-BE cells (D 1.5). Lower panel: RT-qPCR analysis of CyCoNP, miR-4492 and miR-1249-5p transcripts in the specific pull-down (CyCoNP PD) and in the control (LacZ PD) RNA samples. Values are expressed as percentage (%) of Input and represent means ± SEM of three biological replicates. DSS: Dextran Sulfate Sodium Salt; PD: pull-down. (**D**) Left panel: pie chart representing the total number of CyCoNP-downregulated mRNAs and the fraction of miR-4492 *bona fide* targets. Right panel: bar plot showing the distribution of miR-4492 *bona fide* targets in the GO categories previously identified for CyCoNP-downregulated genes (see Figure 1G). (**E**) RT-qPCR quantification of *NCAM1*, *SLC18A3*, *ELMO1*, *PHOX2B*, *CTSV* transcripts in SK-N-BE cells (D 1.5) treated with LNA-SCR or LNA targeting miR-4492. Data were normalized to *GAPDH* transcript and represent means ± SEM of three biological replicates. Data information: ns (non-significant) p>0.05, *P < 0.05, **P < 0.01, unpaired Student's *t* test.

Cytoplasmic lncRNAs have also been illustrated to regulate gene expression post-transcriptionally in many ways by cooperating with different molecules such as miRNAs (62). The association between lncRNAs and miRNAs has been extensively characterized in the last ten years (63–65), however caution must be taken when an interaction with miRNAs is predicted due to their possible promiscuous relationships (66). To initially test in an unbiased manner whether CyCoNP could interact with specific miRNAs in neuronal cells, we selected miRNAs commonly expressed from small RNA-Seq of both hiPSC-derived MN and neuroblastoma samples (42,67) screening for potential binding on CyCoNP sequence using miRanda algorithm (48); Supplementary Figure S2G, Supplementary Table S3). We filtered the list of putative miRNAs that can associate with CyCoNP by selecting the best candidates based on the highest number of binding sites and the best sum energy (Supplementary Figure S2G, Supplementary Table S3). We found at the top of the list hsa-miR-1249-5p and hsa-miR-4492, which display five and three binding sites (MRE), respectively, and good binding energy (Figure 2A, Supplementary Table S3). We then checked the association of the lncRNA with the miRNA-associated machinery by performing an AGO2 Cross-linking and immunoprecipitation assay (CLIP) in SK-N-BE cells. Analysis of the retrieved proteins confirmed the successful precipitation of AGO2 (Supplementary Figure S2H, left panel) while RT-qPCR analysis showed the enrichment of CyCoNP lncRNA in the immunoprecipitated samples as compared to *GAPDH* mRNA and to IgG samples (Supplementary Figure S2H, right panel). Next, we tested whether CyCoNP could be responsive to the two miRNA candidates by establishing a luciferase-based reporter assay cloning the entire CyCoNP sequence downstream of the ORF of Renilla luciferase (Figure 2B, upper panel). We co-transfected this construct in SK-N-BE cells together with LNA molecules targeting hsa-miR-1249-5p, hsa-miR-4492 or a scrambled sequence used as a control (LNA-SCR). Interestingly, cells treated with LNA against miR-4492 exhibited a significant increase in luciferase activity compared to LNA-SCR, while no differences were detected when cells were treated with LNA against hsa-miR-1249-5p (Figure 2B, lower panel). To corroborate these data, we cloned a mutant version of CyCoNP lacking the two regions containing the three putative miR-4492 miRNA responsive elements (MREs, CyCoNP ΔmiR-4492) downstream of the Renilla luciferase open reading frame (ORF). We then co-transfected this construct with LNA-SCR or with LNA targeting miR-4492. In neither case, we observed alteration of the luciferase signal, confirming the specificity of wild type CyCoNP responsiveness to miR-4492 and the reliability of the *in silico* prediction (Supplementary Figure S2I). In order to prove the direct association between these RNA molecules, we set up an endogenous RNA pull-down (PD) assay in SK-N-BE cells, targeting CyCoNP with 20 nt long antisense biotinylated probes designed across the entire sequence, together with the addition of Dextran Sulfate Sodium Salt (DSS) to increase RNA recovery (Figure 2C, upper panel; Supplementary Table S1; 32). RT-qPCR analysis of the retrieved RNA highlighted a significant enrichment of CyCoNP in the PD samples compared to the LacZ control (Figure 2C, lower panel). When miRNAs were analyzed, we found the specific recovery of miR-4492 in the PD fractions, compared to the negative control, while no signal was detected for miR-1249-5p in PD nor in LacZ samples (Figure 2C, lower panel). Altogether, these data indicated that CyCoNP interacts *in vivo* with the AGO protein and with miR-4492.

To investigate if the selected miRNA could participate in the gene regulation mediated by CyCoNP, we screened the list of genes downregulated by the lncRNA depletion searching for *bona fide* miR-4492 targets. By combining the predictions of three algorithms, namely TargetscanV8, miRanda and PITA (50,48,49), we found that among the 373 mRNAs downregulated in si-CyCoNP conditions (Figure 1F; Supplementary Table S2), 77 (20%) resulted *bona fide* miR-4492 targets (Figure 2D left panel, Supplementary Table S3). Interestingly, these targets were mainly distributed among the ‘Neuron differentiation’ category, previously identified for CyCoNP-downregulated genes (Figure 2D right panel). To assess miR-4492 function in SK-N-BE cells, we blocked its activity with antisense LNA molecules and analyzed the expression levels of a subset of putative interesting targets (*NCAM1*, *SLC18A3*, *ELMO1*, *PHOX2B* and *CTSV*). The results indicate that all the analyzed genes display a slight, though significant, increase in their expression values upon treatment with LNA (Figure 2E).

It is important to note that the CyCoNP-dependent regulation of these targets cannot be due to alteration of miR-4492 since its levels are not affected upon CyCoNP knock-down (Supplementary Figure S2J). Altogether, these data suggest that CyCoNP, by binding miR-4492, can affect its activity on a subset of target mRNAs.

### CyCoNP interacts with specific mRNAs and controls NCAM1 expression at different levels

**Figure 3.**
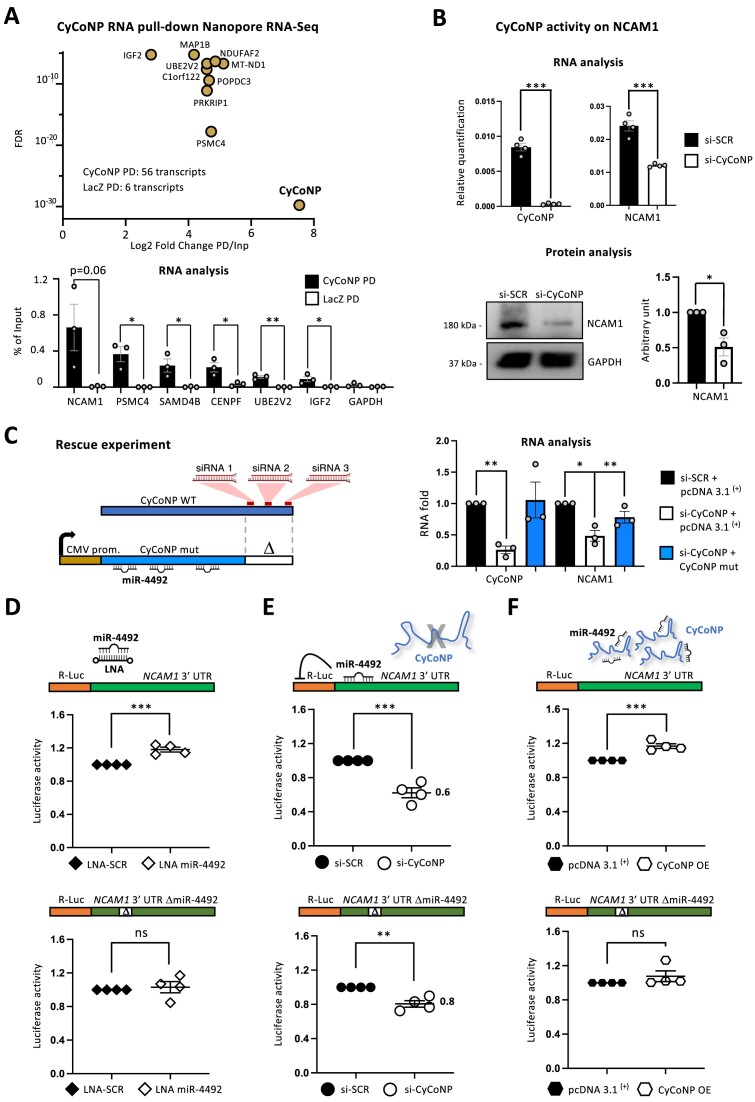
CyCoNP interacts with specific mRNAs and controls NCAM1 expression at different levels. (**A**) Upper panel: scatter plot showing the top 10 enriched transcripts in CyCoNP RNA pull-down RNA-Seq assay. The relative number of transcripts enriched in the specific pull-down (CyCoNP PD) and in the control (LacZ PD) samples are shown. Lower panel: RT-qPCR analysis of *NCAM1*, *PSMC4*, *SAMD4B*, *CENPF*, *UBE2V2* and *IGF2* transcripts in the specific pull-down (CyCoNP PD) and in the control (LacZ PD) RNA samples. *GAPDH* transcript serves as negative control. Values are expressed as percentage (%) of Input and represent means ± SEM of three biological replicates. (**B**) Upper panel: RT-qPCR quantification of CyCoNP and *NCAM1* transcripts in SK-N-BE cells (D 1.5) treated with si-SCR or si-CyCoNP. Data were normalized to *GAPDH* transcript and represent means ± SEM of four biological replicates. Lower panel: representative western blot analysis for NCAM1 in SK-N-BE cells (D 1.5) treated with si-SCR or si-CyCoNP. GAPDH was used as a loading control. Right: quantification of NCAM1 signal intensity relative to GAPDH is shown aside; data represent means ± SEM of three biological replicates. (**C**) Left: schematic representation of the rescue-based construct. The sequence of CyCoNP WT (in dark blue) is shown together with the position of the targeting sites of the three siRNAs used for CyCoNP depletion (marked as red boxes). The sequence of CyCoNP lacking 150 nucleotides at its 3′ end, which is devoid of the three siRNA binding sites (CyCoNP mut, in light blue), was cloned downstream of CMV promoter (brown). The relative position of the predicted miR-4492 binding sites on CyCoNP mut sequence in shown. Right: RT-qPCR quantification of CyCoNP and *NCAM1* mRNA in SK-N-BE cells (D 1.5) co-transfected with si-SCR or si-CyCoNP and with pcDNA 3.1 ^(+)^ empty vector or CyCoNP mut vector. Data were normalized to *GAPDH* transcript and represent means ± SEM of three biological replicates. *P < 0.05, **P < 0.01, paired Student's *t* test. **See Materials and methods for details**. (**D**) Schematic representation of the *NCAM1* luciferase-based reporter constructs. The entire sequence of *NCAM1* 3′ UTR (WT, upper panel) or *NCAM1* 3′ UTR sequence lacking the predicted miR-4492 binding site (ΔmiR-4492, lower panel) were cloned downstream of the Renilla luciferase ORF represented in orange. Quantification of Renilla luciferase activity in SK-N-BE cells co-transfected with the *NCAM1* 3′ UTR (WT, upper panel) or *NCAM1* 3′ UTR ΔmiR-4492 (lower panel) constructs and LNA-SCR or LNA targeting miR-4492. Data represent the mean luciferase activity ± SEM of four biological replicates. See Materials and methods for details. (**E**) Schematic representation of the *NCAM1* luciferase-based reporter constructs. The entire sequence of *NCAM1* 3′ UTR (WT, upper panel) or *NCAM1* 3′ UTR sequence lacking the predicted miR-4492 binding site (ΔmiR-4492, lower panel) was cloned downstream of the Renilla luciferase ORF represented in orange. Quantification of Renilla luciferase activity in SK-N-BE cells co-transfected with the *NCAM1* 3′ UTR (WT, upper panel) or *NCAM1* 3′ UTR ΔmiR-4492 (lower panel) constructs and si-SCR or si-CyCoNP. Data represent the mean luciferase activities ± SEM of four biological replicates. See Materials and methods for details. (**F**) Schematic representation of the *NCAM1* luciferase-based reporter constructs. The entire sequence of *NCAM1* 3′ UTR (WT, upper panel) or *NCAM1* 3′ UTR sequence lacking the predicted miR-4492 binding site (ΔmiR-4492, lower panel) was cloned downstream of the Renilla luciferase ORF represented in orange. Quantification of Renilla luciferase activity in SK-N-BE cells co-transfected with the *NCAM1* 3′ UTR (WT, upper panel) or *NCAM1* 3′ UTR ΔmiR-4492 (lower panel) constructs and pcDNA 3.1 ^(+)^ empty vector or CyCoNP overexpression vector. Data represent the mean luciferase activity ± SEM of four biological replicates. **See Materials and methods for details**. Data information: ns (non-significant) *P* > 0.05, **P < 0.01, ***P < 0.001, unpaired Student's *t* test.

Recent data indicate the propensity of lncRNAs to interact and regulate mRNAs (19), therefore, we integrated the analysis of miRNA interactors with that of mRNA by submitting biological duplicates of CyCoNP RNA pull-down samples performed in SK-N-BE cells to Oxford Nanopore RNA sequencing. Analysis of the sequencing data revealed the significant enrichment of 56 RNAs (Log_2_ Fold Change PD/Inp > 1; FDR < 0.05) in CyCoNP PD samples compared to the LacZ PD control. CyCoNP resulted at the top of the list both for fold enrichment (Log2 Fold Change PD/Inp > 7.5) and statistical significance (FDR < 1.7 × 10^−30^; Figure 3A, upper panel; Supplementary Table S4), confirming the reliability of the RNA pull-down procedure. To further corroborate our data, we validated the enrichment of a subset of interacting targets on additional RNA pull-down biological replicates (Figure 3A, lower panel). Among the list of interacting mRNAs, we found several genes involved in different aspects of neuron development (Supplementary Table S4) and with the aim to focus on specific functional interactions we intersected the list of CyCoNP mRNA binders (Supplementary Table S4) with the list of CyCoNP-downregulated genes (Supplementary Table S2). This analysis led to the identification of *NCAM1* mRNA as a transcript being both downregulated and bound by CyCoNP (Supplementary Figure S3A). Since NCAM1 is known to be a crucial player in many aspects of neuron physiology (68,69,26) we deepened our analysis on this promising candidate. As a first step, we set up a psoralen (AMT, 4′-Aminomethyltrioxsalen hydrochloride) cross-linking RNA pull-down assay of CyCoNP in SK-N-BE cells (Supplementary Figure S3B, upper panel). The psoralen (AMT) compound, by intercalating into nucleic acids, cross-links the direct RNA–RNA interactions in living cells upon irradiation with the Ultraviolet (UV) light (70). Analysis of the purified RNAs, revealed the significant enrichment of CyCoNP in the specific PD samples which was accompanied by the significant recovery of *NCAM1* transcript compared to the LacZ sample and the *GAPDH* mRNA (Supplementary Figure S3B, lower panel). These results indicate that CyCoNP and *NCAM1* mRNA directly interact *in vivo*. Notably, *NCAM1* mRNA was also validated as a miR-4492 target (Figure 2E) with a miRNA binding site consistently predicted by all the implemented softwares (Supplementary Table S3). To assess the functional interplay between the lncRNA and the mRNA we performed CyCoNP depletion using siRNAs in SK-N-BE cells to analyze both RNA and protein extracts. Analyses of the retrieved samples show that the downregulation of the lncRNA expression is paralleled by a significant decrease (50%) of both *NCAM1* mRNA and protein levels (Figure 3B). To confirm the direct responsiveness of *NCAM1* mRNA to CyCoNP, we set up a rescue experiment in SK-N-BE cells by overexpressing a lncRNA mutant version (CyCoNP mut) resistant to siRNA-mediated knock-down (Figure 3C). As a control, we used an empty overexpression vector (pcDNA 3.1 ^(+)^). These two constructs were co-transfected with siRNAs targeting CyCoNP (si-CyCoNP), or with siRNA scrambled controls (si-SCR). In this experiment, we employed siRNAs targeting the endogenous transcript, which are instead unable to target the exogenous mutant CyCoNP (Supplementary Table S1). The results show that while endogenous CyCoNP depletion is accompanied by the downregulation of *NCAM1* mRNA, the overexpression of the siRNA-resistant CyCoNP produced a 30% recovery of *NCAM1* mRNA levels (Figure 3C, right panel). These data highlighted robust and direct relationships between the two molecules but still do not provide any evidence on the putative involvement of miR-4492 in this regulatory mechanism. To test this, we established a luciferase-based reporter assay by cloning the entire sequence of *NCAM1* 3′ UTR or a mutant version lacking the predicted miR-4492 binding region (Supplementary Table S3) downstream of the Renilla Luciferase ORF. We started to assess the responsiveness of these constructs to the miRNA by co-transfecting them in SK-N-BE cells together with an LNA targeting miR-4492 or a scrambled control (Figure 3D). Upon miRNA blocking, we found a significant increase of luciferase activity only for the *NCAM1* 3′ UTR WT construct (Figure 3D, upper panel) while, as expected, no differences were detected for the *NCAM1* 3′ UTR mutant construct (Figure 3D, lower panel). Next, we knocked-down CyCoNP and observed a strong decrease in the luciferase signal for the *NCAM1* 3′ UTR WT construct (Figure 3E, upper panel). Conversely, using *NCAM1* 3′ UTR mutant construct, the effect induced by CyCoNP depletion was much lower (Figure 3E, lower panel). Finally, when we overexpressed CyCoNP, we observed a significant increase of the luciferase activity only in presence of *NCAM1* 3′ UTR WT (Figure 3F, upper panel), while no difference was observed for the *NCAM1* 3′ UTR mutant (Figure 3F, lower panel). Altogether, these analyses revealed that CyCoNP can control *NCAM1* expression through miR-4492. To corroborate this evidence, we set up a luciferase-based reporter assays in murine N2a cells, a model system in which CyCoNP, *NCAM1* mRNA and miR-4492 are not expressed. We co-transfected CyCoNP WT and *NCAM1* 3′ UTR WT with miR-4492 mimics and found for both constructs a significant decrease of the luciferase signal, with CyCoNP displaying a slightly higher responsiveness (Supplementary Figure S3C). These results demonstrate that CyCoNP and *NCAM1* mRNA respond in a comparable manner to the binding of hsa-miR-4492.

Given the central role of NCAM1 in supervising neuron differentiation and cell development gene expression programs, we wondered whether the downregulation of genes belonging to these biological categories observed upon CyCoNP depletion (Figure 1G, Supplementary Table S2) could, at least in part, be explained as a consequence of CyCoNP-dependent NCAM1 deficiency. To verify this, we silenced *NCAM1* mRNA expression by performing RNA interference in SK-N-BE cells using a pool of four different siRNAs (Supplementary Table S1). RT-qPCR analysis shows that *NCAM1* mRNA depletion parallels the concomitant downregulation of a subset of targets chosen from the list of CyCoNP-downregulated genes according to their biological relevance (Supplementary Table S2, Supplementary Figure S3D). Notably, the analyzed mRNAs are not predicted targets of miR-4492 (Figure 2D, Supplementary Table S3), suggesting a double mechanism of gene expression regulation mediated by CyCoNP: one related to the mechanism of miR-4492 sponge on given targets, and the other accounting for the precise control of *NCAM1* mRNA expression and in turn to its downstream targets.

### Functional direct RNA–RNA interactions between CyCoNP and *NCAM1* mRNA

**Figure 4.**
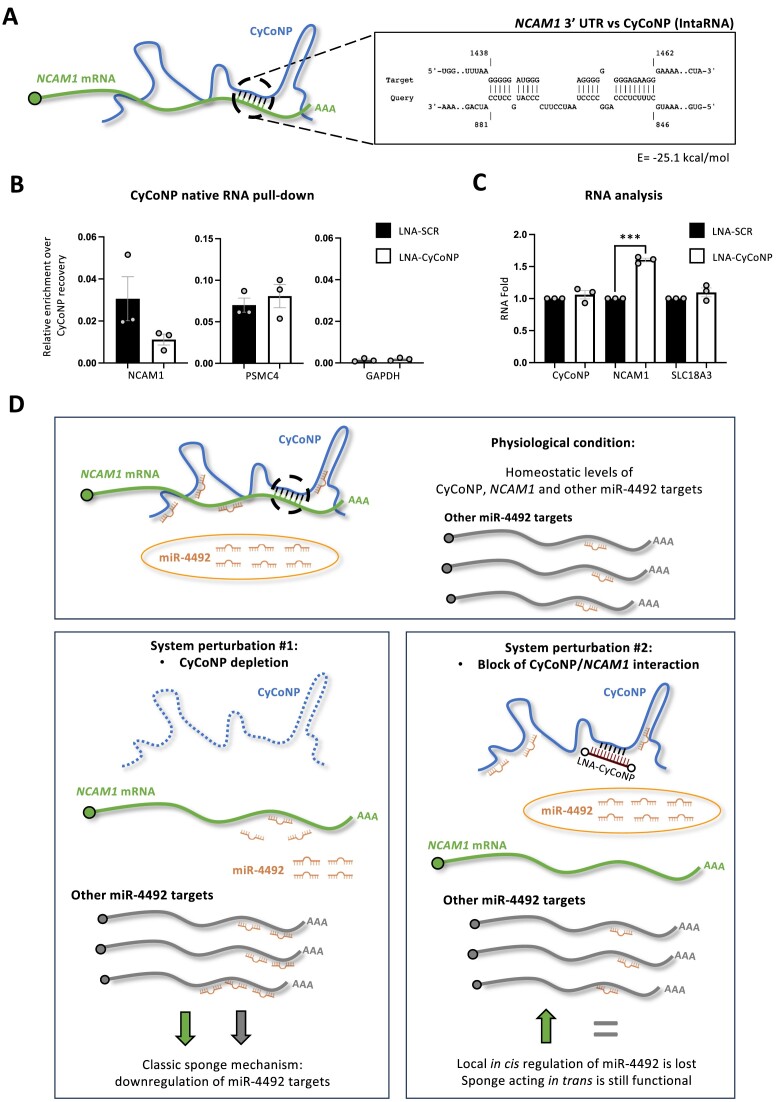
Functional direct RNA–RNA interactions between CyCoNP and *NCAM1* mRNA. (**A**) Schematic representation of CyCoNP and *NCAM1* transcripts. The best predicted region of interaction between the two molecules is highlighted in black. The energy (*E*) of interaction retrieved by IntaRNA is shown. (**B**) RT-qPCR analysis of *NCAM1* and *PSMC4* transcripts in CyCoNP PD RNA samples from SK-N-BE cells (D 1.5) treated with LNA-SCR or LNA-CyCoNP. *GAPDH* transcript serves as a negative control. Values are expressed as relative enrichment normalized over the % of CyCoNP transcript precipitated in each condition and represent means ± SEM of three biological replicates. (**C**) RT-qPCR analysis of CyCoNP, *NCAM1* and *SLC18A3* transcripts in SK-N-BE cells (D 1.5) treated with LNA-SCR or LNA-CyCoNP. Data were normalized to *GAPDH* transcript and represent means ± SEM of three biological replicates. (**D**) Proposed model for CyCoNP mechanism of action: in physiological conditions (upper panel), CyCoNP is highly expressed in the cytoplasm of neuronal cells where it acts as a miR-4492 molecular sponge and, synergically, it establishes direct RNA–RNA interactions with *NCAM1* mRNA to ensure the exact loading of the miRNA on this transcript. The proper functioning of all these components allows the homeostatic expression of CyCoNP, NCAM1 and the others miR-4492 targets. By depleting the lncRNA expression (left panel), CyCoNP activity as an abundant sponge is completely lost resulting in the overall downregulation of miR-4492 targets, including NCAM1. By hampering the interaction between CyCoNP and *NCAM1* mRNA (right panel), without altering the lncRNA abundance, CyCoNP *in trans* regulation as a miR-4492 sponge is preserved while the *in cis* regulation as a facilitator of miR-4492 loading on *NCAM1* mRNA is lost. Indeed, the inhibition of the RNA–RNA interaction alters the local concentration of miR-4492 on *NCAM1* mRNA and, as a direct consequence, changes its expression levels. Data information: ***P < 0.001, unpaired Student's *t* test.

As revealed by the psoralen-based RNA pull-down assay, CyCoNP establishes direct interactions with *NCAM1* mRNA in SK-N-BE cells (Supplementary Figure S3B). To dig into this molecular *liaison*, we selected the best predicted region of interaction between the CyCoNP and the *NCAM1* 3′ UTR sequences, as computed by IntaRNA (71; Figure 4A) to design an antisense LNA molecule against CyCoNP that could prevent its interaction with *NCAM1* mRNA (LNA-CyCoNP; Supplementary Figure S4A). Notably, by transfecting SK-N-BE cells with LNA-CyCoNP, we were indeed able to block the interaction between the lncRNA and the mRNA *in vivo* as demonstrated by a three-fold diminished recovery of *NCAM1* mRNA in the CyCoNP RNA pull-down compared to a control transfection with a scrambled LNA (Figure 4B). Instead, no differences in enrichment recovery were found between the two conditions for another CyCoNP interactor, *PSMC4* mRNA, predicted to bind CyCoNP in a different portion of the lncRNA (Supplementary Figure S4B), as well as for a non-interacting control mRNA, *GAPDH* (Figure 4B). To note, LNA-CyCoNP transfection does not affect CyCoNP expression, as well as that of *SLC18A3* mRNA (Figure 4C), a miR-4492 target whose expression is controlled by the lncRNA (Figure 2E, Supplementary Figure S1F; Supplementary Table S2; Supplementary Table S3). Conversely, upon the same treatment, we observed a significant increase of *NCAM1* mRNA levels (Figure 4C), suggesting a specific and local regulation exerted by CyCoNP on the *NCAM1* mRNA. To test whether this regulation could specifically depend on the availability of miR-4492 on *NCAM1* mRNA, we set up a luciferase-based reporter assay by exploiting the *NCAM1* 3′ UTR WT and *NCAM1* 3′ UTR ΔmiR-4492 constructs. By transfecting LNA-CyCoNP, we observed a significant two-fold increase in the signal generated by the *NCAM1* 3′ UTR WT luciferase construct compared to the control (LNA-SCR; Supplementary Figure S4C, left panel). Strikingly when the same experiment was performed using the *NCAM1* 3′ UTR ΔmiR-4492 construct, which is insensible to miR-4492 (Figure 3D), we found that LNA-CyCoNP transfection did not induce any alteration of the luciferase signal compared to the control (Supplementary Figure S4C, right panel). These results highlight a specific mechanism of *NCAM1* mRNA regulation mediated by the physical interaction between CyCoNP and *NCAM1* 3′ UTR, that influences the capability of miR-4492 to repress its target mRNA. Importantly, this effect cannot be ascribed to alterations in miR-4492 levels since they do not change upon LNA-CyCoNP treatment (Supplementary Figure S4D), but instead it appears to be due to a facilitating activity of CyCoNP on miR-4492 for the repression of *NCAM1* mRNA. From these data, we suggest that CyCoNP acts both *in trans* and *in cis*: *in trans* it functions as a classical sponge by competing for the binding of miR-4492 to its mRNA targets, including *NCAM1* mRNA, as evidenced by the overall downregulation of the miR-4492 targets upon its depletion. On the other hand, CyCoNP lncRNA acts *in cis* as an enhancer of miRNA activity by favoring the interaction of miR-4492 with *NCAM1* mRNA, as evidenced by the specific up-regulation of *NCAM1* mRNA only when the pairing with the lncRNA is prevented. This effect could be likely due to the increase in local miR-4492 concentration around the *NCAM1* mRNA. These features suggest a model according to which CyCoNP depletion would repress the levels of the miR-4492 targets, while disrupting the pairing with *NCAM1* mRNA would specifically affect miRNA loading on *NCAM1* mRNA (Figure 4D).

### CyCoNP depletion impinges on neuronal physiology

**Figure 5.**
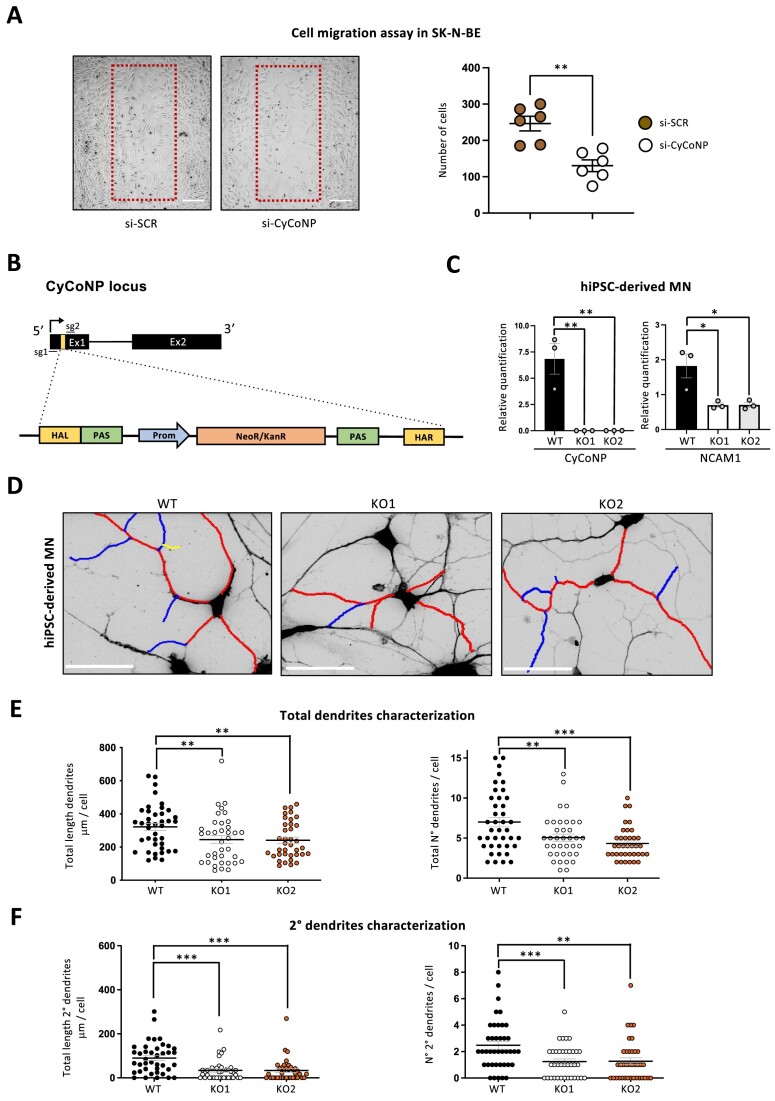
CyCoNP depletion impinges on neuronal physiology. (**A**) Left: representative image of SK-N-BE cells (D 1.5) treated with si-SCR or si-CyCoNP after 24h of cell scratching. Red boxes highlight the region of interest (ROI) representing the wound area. White lines represent scale bars corresponding to 100 μm. Right: quantification of SK-N-BE cells (D 1.5) treated with si-SCR or si-CyCoNP that migrate in the ROI after 24h of cells scratching and starvation. Each dot represents the counted cells for each acquisition (2 images for each biological replicate). Data represent mean ± SEM of three biological replicates. (**B**) Schematic representation of the genome-editing strategy to block CyCoNP transcription in hiPSCs. The donor vector used for the homology recombination is shown. HAL and HAR: Left and Right Homology Arms, PAS: Poly Adenilation Signal, Prom: Promoter, NeoR/KanR: Neomycin/Kanamycin resistance cassette. The relative position of the two single guide RNAs (sg1 and 2; sgRNAs) co-transfected to target CyCoNP exon 1 is shown. **See Materials and methods for details**. (**C)** RT-qPCR analysis of CyCoNP (left) and *NCAM1* (right) transcripts in hiPSC-derived MN at day 8 of differentiation in CyCoNP wild-type (WT) cells and two different CyCoNP knock-out cellular clones (KO1, KO2). Data were normalized to *ATP5O* transcript and represent means ± SEM of three biological replicates. (**D**) Representative confocal captions showing tracing analysis performed on CyCoNP WT, KO1 and KO2 hIPSC-derived MN selected in an unbiased way. Dendrites were labelled as primary (red), secondary (blue) and tertiary (yellow) on inverted coloured fluorescent images. White lines represent scale bars corresponding to 50 μm. (**E**) Left: dot plot representing the values distribution of elongation for sum length of total branches (total length of dendrites per cell) in CyCoNP WT, KO1 and KO2 hIPSC-derived MN. Right: dot plot representing the total number of dendrites per cell. A total of #40, #37, #37 cells were traced for each condition Data represent mean ± SEM of three biological replicates. (**F**) Left: dot plot representing the values distribution of elongation for sum length of all secondary branches (total length of 2° dendrites per cell) in CyCoNP WT, KO1 and KO2 hIPSC-derived MN. Right: dot plot representing the number of 2° dendrites per cell. A total of #40, #37, #37 cells were traced for each condition. Data represent mean ± SEM of three biological replicates. Data information: *P < 0.05, **P < 0.01, ***P < 0.001 unpaired Student's *t* test.

Over the years, many studies have contributed to identifying NCAM1 as a prominent factor involved in different steps of neurogenesis and, particularly, it is associated to the control of cell-to-cell adhesion, cell migration and neurite outgrowth (23,72,28). Since we found NCAM1 expression being highly CyCoNP-dependent, we wondered whether the depletion of the lncRNA in SK-N-BE cells could lead to a phenotypic alteration inherent to the processes supervised by its main target. We then set up cell migration assays on SK-N-BE cells by performing scratch-wound experiments. Upon CyCoNP depletion, we found a significant decrease in the cell migration capability compared to the control treatment (Figure 5A, left panel). Indeed, by counting the cells that populate the wound area after 24 h of starvation, we observed an almost 2-fold decrease in cell number in condition of si-CyCoNP compared to the control (Figure 5A, right panel). On the other hand, the opposite result was observed when we overexpressed *NCAM1* coding sequences (CDS) in SK-N-BE cells (Supplementary Figure S5A). In line with these results, we observed that treatment with LNA against miR-4492 produced an increase in cell migration (Supplementary Figure S5B). Altogether these findings indicate that CyCoNP controls cell migration through miR-4492-dependent NCAM1 regulation.

On this basis and to clarify the direct relationships between CyCoNP and NCAM1 in a more physiological system, we then established a CRISPR/CAS9 genome editing strategy to curb the lncRNA transcription in hiPSCs. To minimize the side effect of genome manipulation that could be due to the deletion of regulatory DNA elements, we inserted a strong polyadenylation site at the beginning of CyCoNP exon 1 that allows the block of transcription without removing any genomic sequence that could bear a regulative function *per se* (Figure 5B; 31). We screened transfected cells by selecting positive clones based on the presence of Neomycin/Kanamycin resistance cassette and selected two putative CyCoNP knock-out (KO) homozygous clones through PCR analysis (Supplementary Figure S5C). We then differentiated the isogenic WT and KO clones towards MN fate and harvested RNA samples after 8 days of differentiation, corresponding to the peak of expression of the lncRNA (Figure 1D, upper panel). RT-qPCR analysis shows that CyCoNP expression is completely abolished in both KO clones (Figure 5C, left panel) and that its depletion is accompanied by a strong and significant downregulation of *NCAM1* mRNA also in this cellular system (Figure 5C, right panel). Interestingly we found that the same subset of genes downregulated in SK-N-BE cells and target of miR-4492 (*SLC18A3*, *ELMO1*, *CTSV* mRNAs) were also expressed at lower levels in CyCoNP KO clones (Supplementary Figure S5D). Also in this case, this downregulation could not be ascribed to differences in miR-4492 total levels, as no significant variation was observed in MN of WT and CyCoNP KO clones (Supplementary Figure S5E). In order to test whether indeed the abundance of CyCoNP could reflect a possible competing endogenous RNA activity, RNA-seq data originated by hiPSC-derived MN (42) were re-analyzed. We found 347 FPKM for CyCoNP and only 52 for *NCAM1* mRNA (Supplementary Table S3). At this time point, when MN are not terminally differentiated and neuronal precursors are present, CyCoNP reaches the highest values and also the miR-4492 targets are upregulated. This, together with the fact that CyCoNP contains three MREs for miR-4492 while *NCAM1* mRNA contains only one, indicates that the stoichiometry goes in the expected direction, with a large excess of CyCoNP. In SK-N-BE cells, the numbers for CyCoNP molecules are lower, as expected for a differentiation system devoid of neuronal progenitors. However, considering the normalization for the MREs number, the two RNAs are in approximate equimolar amounts (15 FPKM x3MREs of CyCoNP versus 50 FPKM x1MRE of *NCAM1* mRNA; Supplementary Table S3).

We then performed phenotypic analysis to assess if CyCoNP depletion could impinge on the differentiation and morphology of hiPSC-derived MN. By performing Immunofluorescence (IF) analysis at day 12 of differentiation, we found that the number of cells positive to MAP2 and Islet1/2 markers remained invariant between WT and KO clones, indicating that the efficiency of the differentiation process is not altered when CyCoNP is depleted (Supplementary Figure S5F, G). Next, we characterized the composition of the dendrites generated in WT and KO hiPSC-derived MN by labeling cells with the dendrites marker MAP2 and with the mature MN marker ChAT (Figure 5D; Supplementary Figure S5H). Interestingly, we found that the total length and number of the dendrites was significantly reduced in both KO clones compared to the WT cells (Figure 5E). In detail, by analyzing the different classes of fibers, we noticed that the length and the number of primary dendrites remained the same between the two conditions (Supplementary Figure S5I) while a strong reduction of these parameters for secondary, tertiary and quaternary dendrites was observed (Figure 5F, Supplementary Figure S5J). These data are particularly relevant in light of the known role of NCAM1 in neuronal branching control (29), reinforcing the importance of the CyCoNP-mediated regulation also in hiPSC-derived MN.

## Discussion

LncRNAs, due to their high versatility, have been described to be involved in almost every aspect of mammalian biology, from the control of specific cellular activities to supervising tissue development processes (73,74). In this study, we have detailed the molecular and biological function of CyCoNP, a lncRNA that we found to be highly expressed in human neural progenitor cells and mostly localized in the cytoplasm. In fact, we describe a clear post-transcriptional control based on direct RNA–RNA interactions, and excluded a regulation at its transcription locus since we found that the proximal HOXC cluster was not deregulated upon CyCoNP depletion (Supplementary Table S2). In detail, we have unraveled a previously unknown, dual mechanism of action that mainly involves miR-4492-mediated regulation of NCAM1, a key regulator of neuronal processes. According to our results, the lncRNA can directly interact with miR-4492 and one of its mRNA targets, *NCAM1*, and in doing so it could locally facilitate the miRNA repressive activity. This appears as a novel function that allows a lncRNA to act as a platform by tethering together a specific miRNA and its target, creating functional RNP complexes and ensuring their efficient colocalization. On the other side, CyCoNP abundance, together with the presence of multiple miR-4492 MREs, make it an effective competing endogenous RNA able to control a set of miR-4492 targets. Specifically, the stoichiometric ratios that we found between CyCoNP and *NCAM1* mRNA favor the idea of a competition for miR-4492 occurring both in hiPSC-derived MN, where CyCoNP is greatly abundant compared to *NCAM1* mRNA, and in SK-N-BE cells, where the two transcripts are roughly in equimolar quantity.

Our data also provide clear and robust evidence of the contribution of CyCoNP lncRNA in the context of neuron physiology and highlight the relevance of its direct control on NCAM1 expression as a paradigmatic example of how RNA–RNA interactions can shape and model specific aspects of cellular homeostasis. Indeed, CyCoNP depletion and the consequent downregulation of NCAM1 produced quite a clear phenotype of neurite branching in hiPSC-derived MN which very well fits with the known role of NCAM1 in triggering intracellular signaling events resulting in cellular responses such as neurite outgrowth (29).

This, and all the molecular mechanisms that over the years have been ascribed to the ever-growing class of lncRNAs, results of particular interest not only as an illustrative example of how noncoding RNAs can exert pleiotropic functions to finely tune gene expression, but also how they may change activity in different cell types. Indeed, CyCoNP (linc-02381) was previously described also in non-neuronal cells and specifically in different kinds of cancer tissues, such as glioma (75), breast (76) and cervical cancers (77). In these studies, it was shown to be a miRNA binder, confirming its ability to interact with the miRNA-containing machinery also in other contexts but with different types of miRNAs and diverse downstream targets.

Keeping the focus on the study of the multiple modes of action of noncoding RNAs could be crucial to elucidate novel mechanisms through which RNA works, thus expanding the understanding of the complex network of regulatory processes at the basis of gene expression control. In particular, the tethering activity of noncoding RNAs is attracting more and more interest due to the importance that these molecules have in controlling the assembly of functional RNPs, where different components can be brought closer together ensuring the efficient execution of biochemical processes.

## Supplementary Material

gkae590_Supplemental_Files

## Data Availability

Sequencing data has been deposited in GEO under accession number GSE245462 (https://www.ncbi.nlm.nih.gov/geo/query/acc.cgi?acc=GSE245462).
