# Supplementary material for: CyCoNP lncRNA establishes cis and trans RNA–RNA interactions to supervise neuron physiology
Source: Nucleic Acids Res. 2024 Jul 11;52(16):9936–52. doi: 10.1093/nar/gkae590 (PMC11381359; doi:10.1093/nar/gkae590)
Supplement: gkae590_Supplemental_Files [file gkae590_supplemental_files.zip › New Supplementary figures proofread.pdf]

Supplementary Figure 1

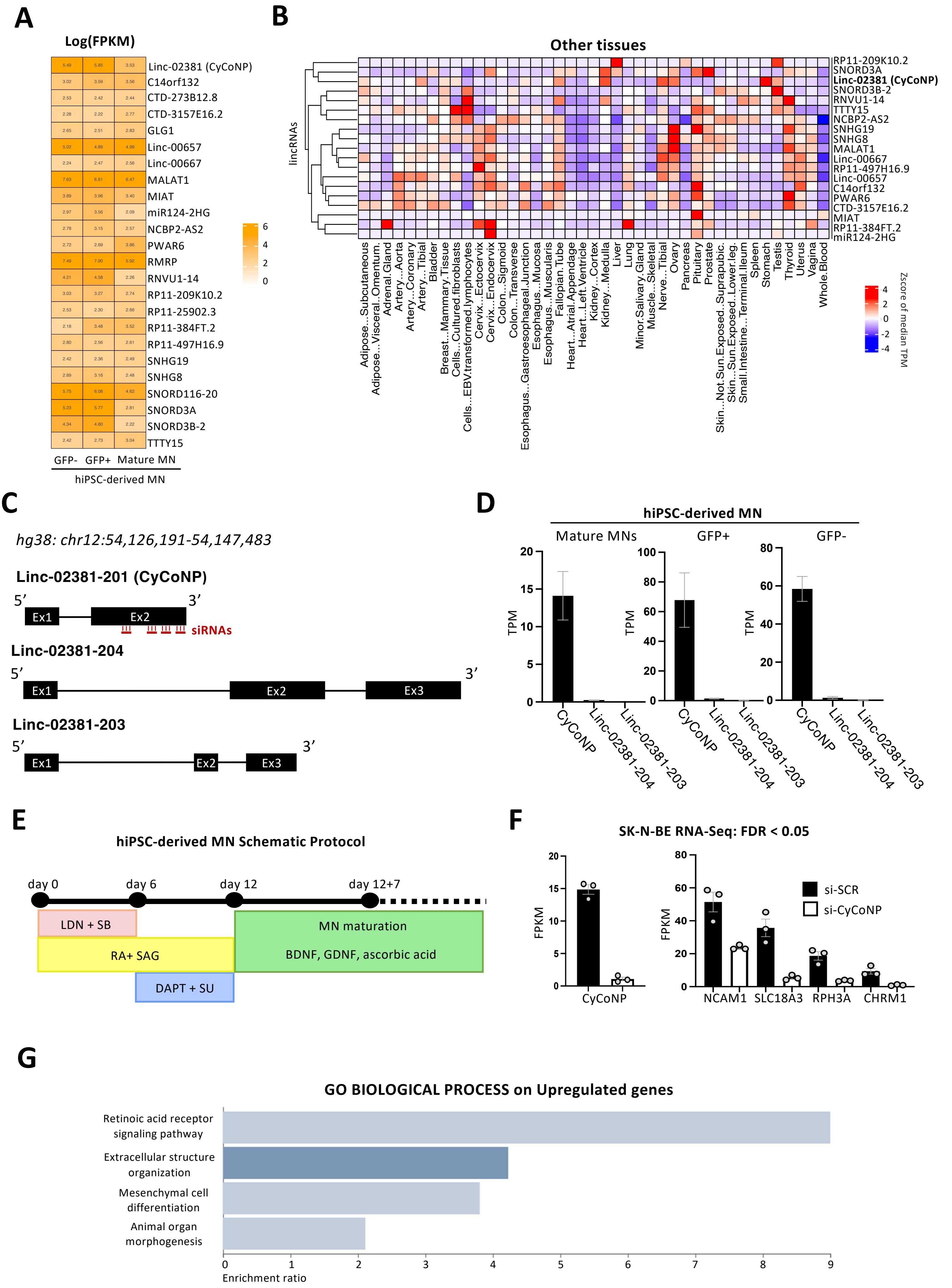

Supplementary Figure 1

A) Heatmap displaying the log(FPKM) of the selected top commonly expressed lincRNAs in MN mix, GFP+ and GFP- cells. FPKM: Fragments Per Kilobase of transcript per Million mapped reads. FDR (False Discovery Rate)

B) Heatmap showing the enrichment of the selected top commonly expressed lincRNAs in MN mix, GFP+ and GFP- cells among the different tissues except brain (other tissues) as retrieved from GTEx portal. Only lincRNAs with available gene ID on GTEx portal are shown.

C) Schematic representation of Linc-02381 locus structure. The relative position of the siRNAs used for CyCoNP (Linc-02381-201) depletion is shown in red. The genomic coordinates according to the latest assembly (*hg 38*) are shown.

D) Expression value, represented as TPM, of the main linc-02381 isoforms in MN mix, GFP+ and GFP- cells. n= three biological replicates

E) Schematic representation of hiPSC-derived MN differentiation protocol. The molecules and chemical compounds added at each step of MN differentiation are indicated. See **Materials and Methods** for details.

F) Expression value, represented as FPKM of CyCoNP (left panel) and *NCAM1*, *SLC18A3*, *RPH3A* and *CHRM1* transcripts (right panel) from the RNA-Seq data in SK-N-BE cells (D 1.5) treated with si-SCR or si-CyCoNP. All represented genes display a FDR (False Discovery Rate) < 0.05. Statistical significance was assessed using the likelihood ratio test (edgeR). See **Materials and Methods** for details.

G) Gene Ontology (GO) enrichment analysis performed by WEBGESTALT (http://www.webgestalt.org) on upregulated genes in SK-N-BE cells (D 1.5) treated with si-CyCoNP compared to si-SCR. Bars indicate the top categories of Biological processes in decreasing order of enrichment ratio. Only the extracellular structure organization category shows an FDR value <0.05.

Supplementary Figure 2

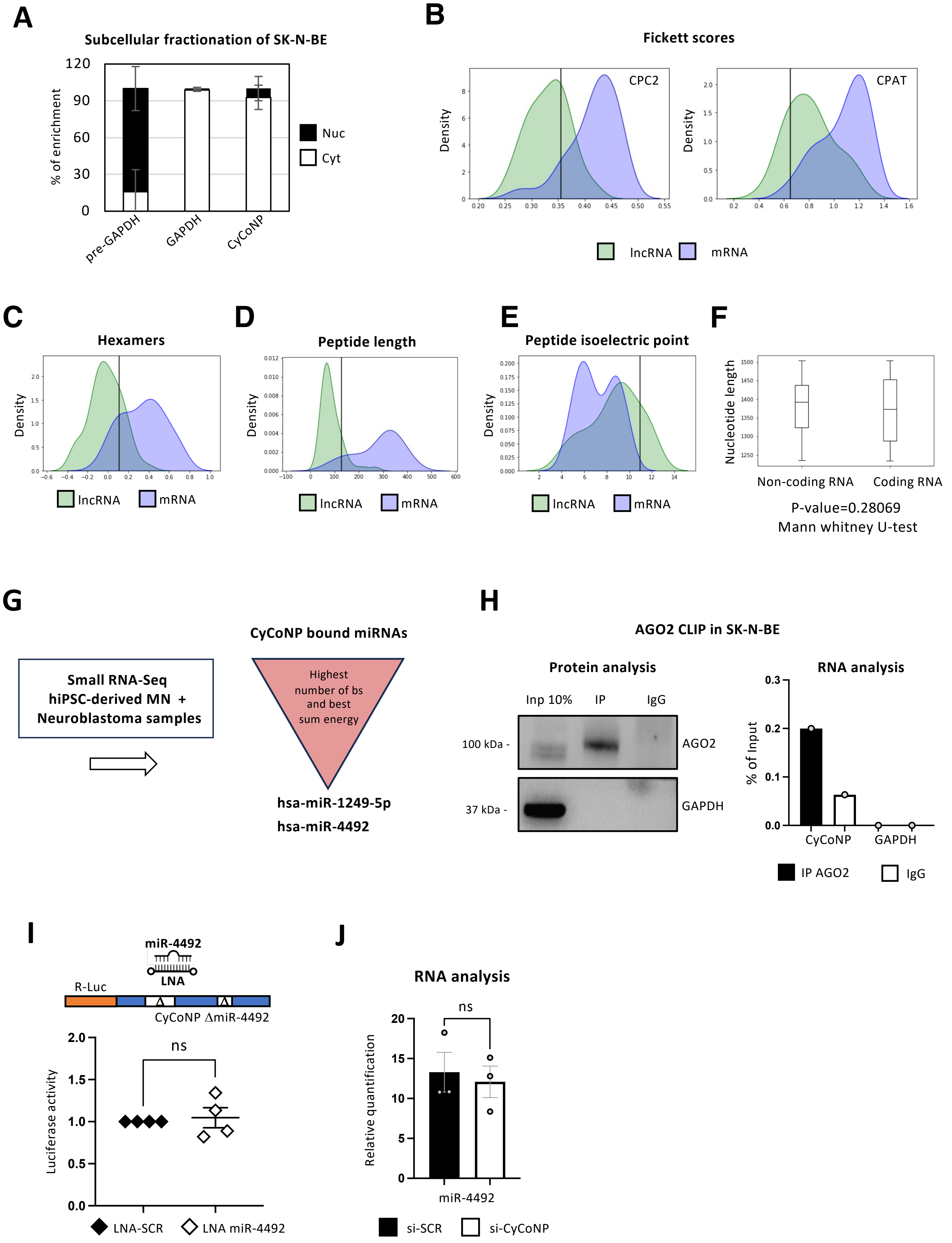

Supplementary Figure 2

A) Quantification of the subcellular distribution of CyCoNP transcript in SK-N-BE cells (D 1.5). The histogram shows the quantification by RT-qPCR of the RNA abundance (%) in cytoplasmic versus nuclear compartments. *GAPDH* and *pre-GAPDH* transcripts were used, respectively, as cytoplasmic and nuclear controls. Data represent mean ± SD of two biological replicates.

B) Kernel density distribution of Fickett scores calculated by CPC2 software (left panel) or CPAT software (right panel) in lncRNAs (green distribution) and mRNAs (blue distribution) belonging to the control sets. A black vertical line highlights the metric value for CyCoNP transcript.

C) Kernel density distribution of the hexamer composition in lncRNAs (green distribution) and mRNAs (blue distribution) belonging to the control sets. A black vertical line highlights the metric value for CyCoNP transcript.

D) Kernel density distribution of the predicted peptide lenght in lncRNAs (green distribution) and mRNAs (blue distribution) belonging to the control sets. A black vertical line highlights the metric value for CyCoNP transcript.

E) Kernel density distribution of the isoelectric point in lncRNAs (green distribution) and mRNAs (blue distribution) belonging to the control sets. A black vertical line highlights the metric value for CyCoNP transcript.

F) Boxplots represent the RNA length (nucleotide) distributions of lncRNAs and mRNAs belonging to the control sets.

G) Computational workflow applied to identify the top two miRNA predicted to interact with CyCoNP.

H) Left: AGO2 western blot analysis on the retrieved protein fractions from AGO2 Cross-linking immunoprecipitation (CLIP) in SK-N-BE cells (D 1.5) in IP and IgG samples. GAPDH protein serves as a loading control. Input (Inp) samples represent 10% of the total protein extracts. Right: RT-qPCR quantification of CyCoNP transcript recovery in AGO2 IP and IgG samples. *GAPDH* transcript serves as negative control. Values are expressed as percentage (%) of Input. n= 1 biological replicate.

I) Upper panel: schematic representation of the CyCoNP ΔmiR-4492 luciferase-based reporter construct. The sequence of CyCoNP lacking two regions containing the three miR-4492 bs was cloned downstream of the Renilla luciferase ORF (orange). See **Materials and Methods** for details. Lower panel: quantification of Renilla luciferase activity in SK-N-BE cells co-transfected with the CyCoNP ΔmiR-4492 luciferase construct and LNA-SCR or LNAs targeting miR-4492. Data represent the mean luciferase activities ± SEM of four biological replicates.

J) RT-qPCR analysis of miR-4492 expression in SK-N-BE cells (D 1.5) treated with si-SCR or si-CyCoNP. Data were normalized over the snRNA U6 transcript and represent means ± SEM of three biological replicates.

Data information: ns (non-significant) $p>0.05$, unpaired Student's t test.

Supplementary Figure 3

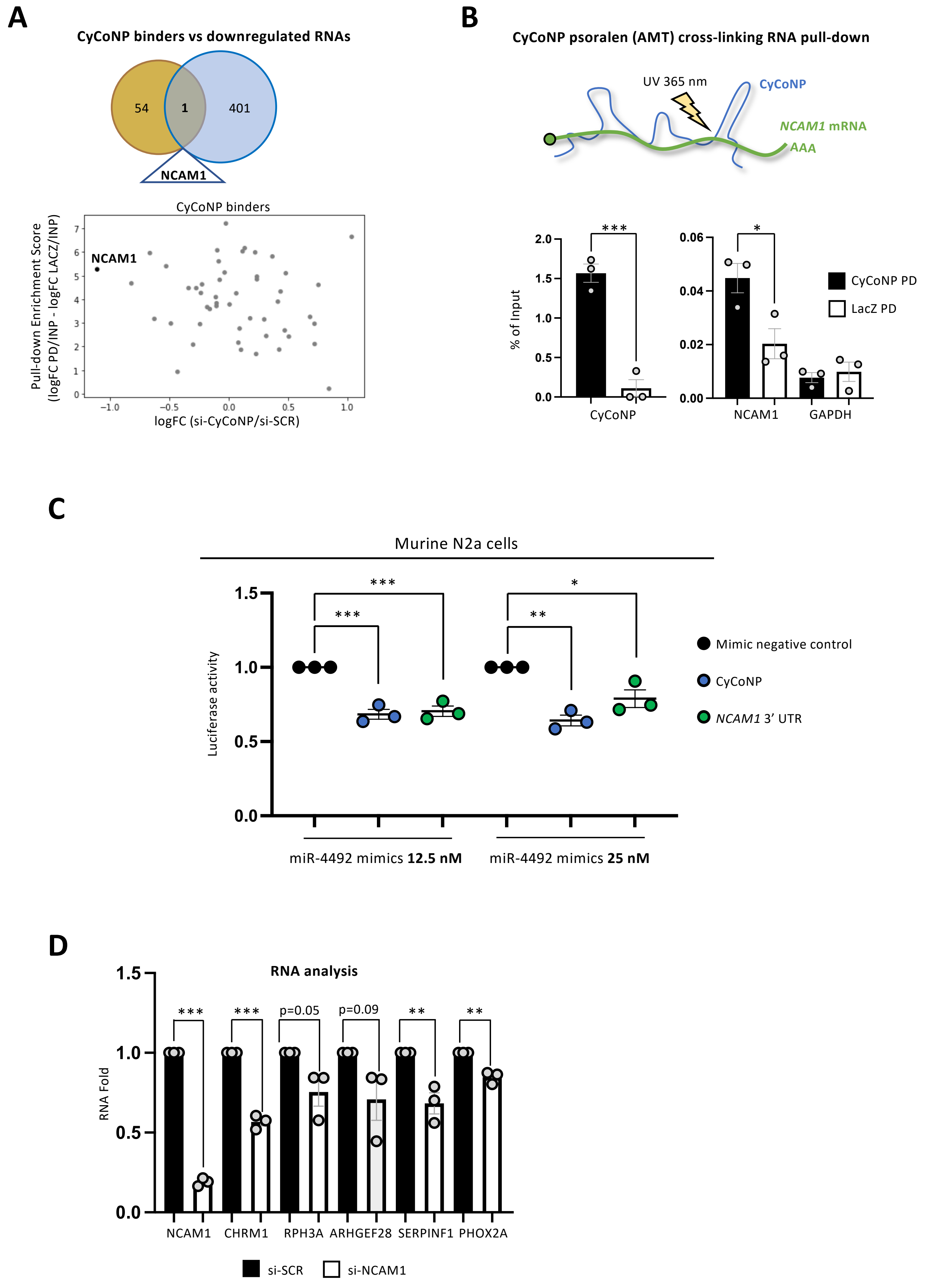

Supplementary Figure 3

A) Upper panel: Venn diagram depicting the intersection of the transcripts enriched in the CyCoNP RNA PD and the down-regulated genes in SK-N-BE cells (D 1.5) treated with si-SCR or si-CyCoNP. Lower panel: Scatterplot illustrating the relationship between CyCoNP RNA pull-down enrichment and the deregulation observed upon CyCoNP depletion for CyCoNP interactors. The extent of deregulation was measured as logFC (si-CyCoNP/SCR) in RNA-Seq experiment while CyCoNP pull-down enrichment was measured as enrichment scores (logFC PD/INP) difference between CyCoNP pull-down and LACZ control.

B) Upper panel: schematic representation of CyCoNP psoralen (AMT)-Cross-linked RNA pull-down workflow from SK-N-BE cells (D 1.5). See **Materials and Methods** for details. PD: pull-down. Lower panel: RT-qPCR analysis of CyCoNP and *NCAM1* transcripts in the specific pull-down (CyCoNP PD) and in the control (LacZ PD) RNA samples. *GAPDH* transcript serves as a negative control. Values are expressed as percentage (%) of Input and represent means ± SEM of three biological replicates.

C) Quantification of Renilla luciferase activity in N2a cells co-transfected with CyCoNP or *NCAM1* 3' UTR WT constructs and 12.5 nM (left panel) or 25 nM (right panel) of mimic negative control or miR-4492 mimics . Data represent the mean luciferase activity ± SEM of three biological replicates. See **Materials and Methods** for details.

D) RT-qPCR quantification of *NCAM1*, *CHRM1*, *RPH3A*, *ARHGEF28*, *SERPINF1* and *PHOX2A* transcripts in SK-N-BE cells (D 1.5) treated with si-SCR or si-NCAM1. Data were normalized to *GAPDH* transcript and represent means ± SEM of three biological replicates.

Data information: *$p < 0.05$, **$p<0.01$, ***$p < 0.001$, unpaired Student's t test.

Supplementary Figure 4

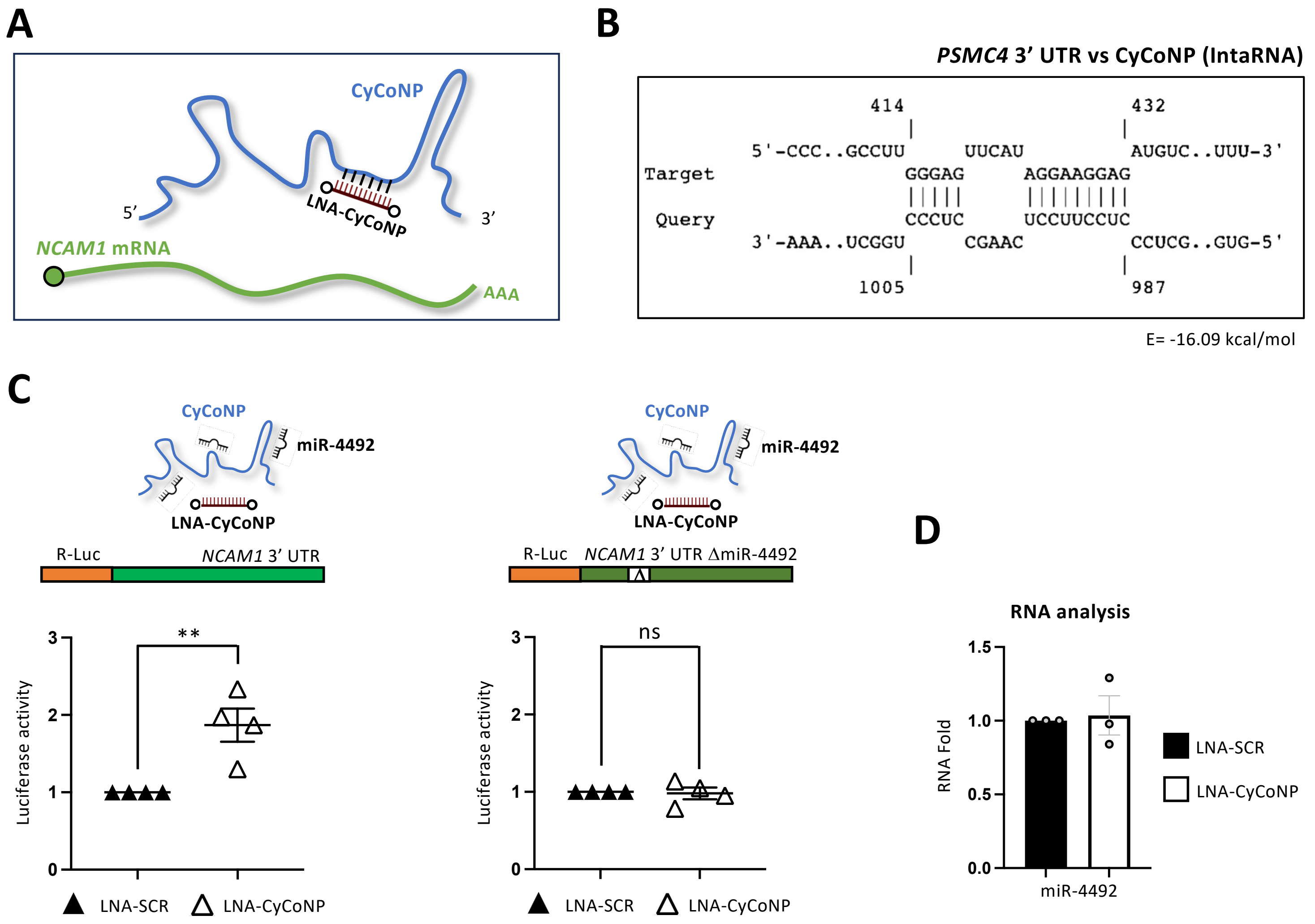

Supplementary Figure 4

A) Schematic representation of CyCoNP and *NCAM1* transcripts. The LNA molecule (LNA-CyCoNP) used to hamper the interaction between the two molecules is shown.

B) Schematic representation of the best predicted region of interaction between CyCoNP and *PSMC4* 3' UTR sequences. The energy (E) of interaction retrieved by IntaRNA is shown.

C) Upper panels: schematic representation of the *NCAM1* luciferase-based reporter constructs. The entire sequence of *NCAM1* 3' UTR (WT, left panel) or *NCAM1* 3' UTR sequence lacking the predicted miR-4492 binding site (ΔmiR-4492, right panel) were cloned downstream of the Renilla luciferase ORF represented in orange. Lower panels: quantification of Renilla luciferase activity in SK-N-BE cells co-transfected with the *NCAM1* 3' UTR (left panel) or *NCAM1* 3' UTR ΔmiR-4492 (right panel) constructs and LNA-SCR or LNA-CyCoNP. Data represent the mean luciferase activity ± SEM of four biological replicates. See **Materials and Methods** for details.

D) RT-qPCR analysis of miR-4492 expression in SK-N-BE cells (D 1.5) treated with LNA-SCR or LNA-CyCoNP. Data were normalized over the snRNA U6 transcript and represent means ± SEM of three biological replicates.

Data information: ns (non-significant) $p>0.05$, $^{**}p<0.01$, unpaired Student's t test.

Supplementary Figure 5

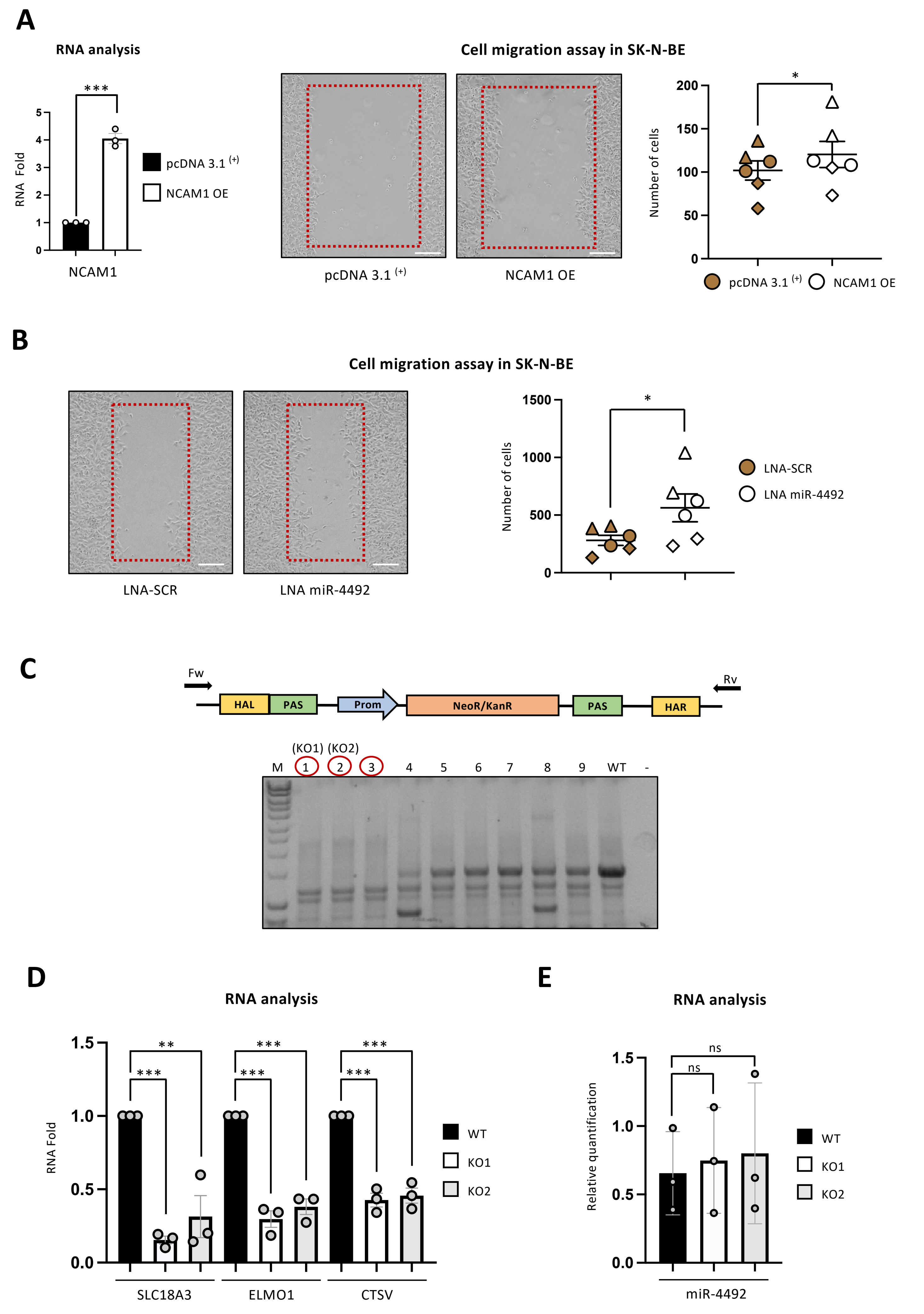

Supplementary Figure 5

F

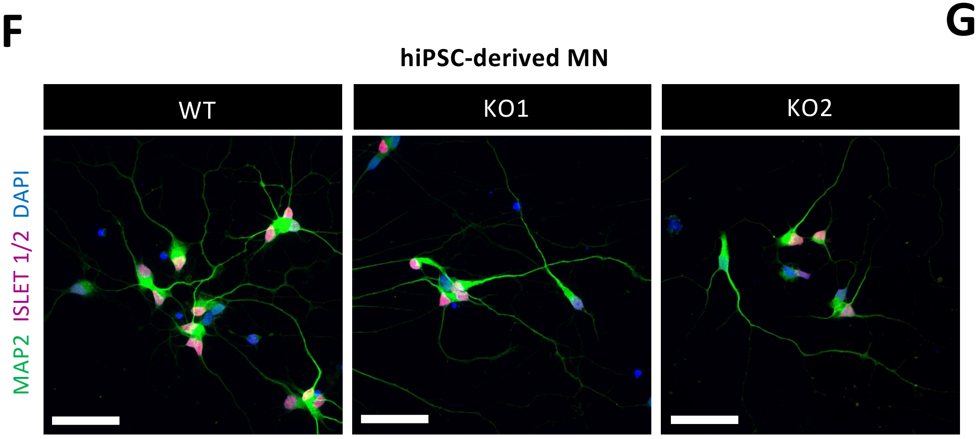

G

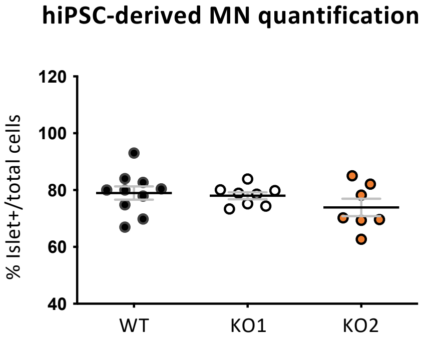

H

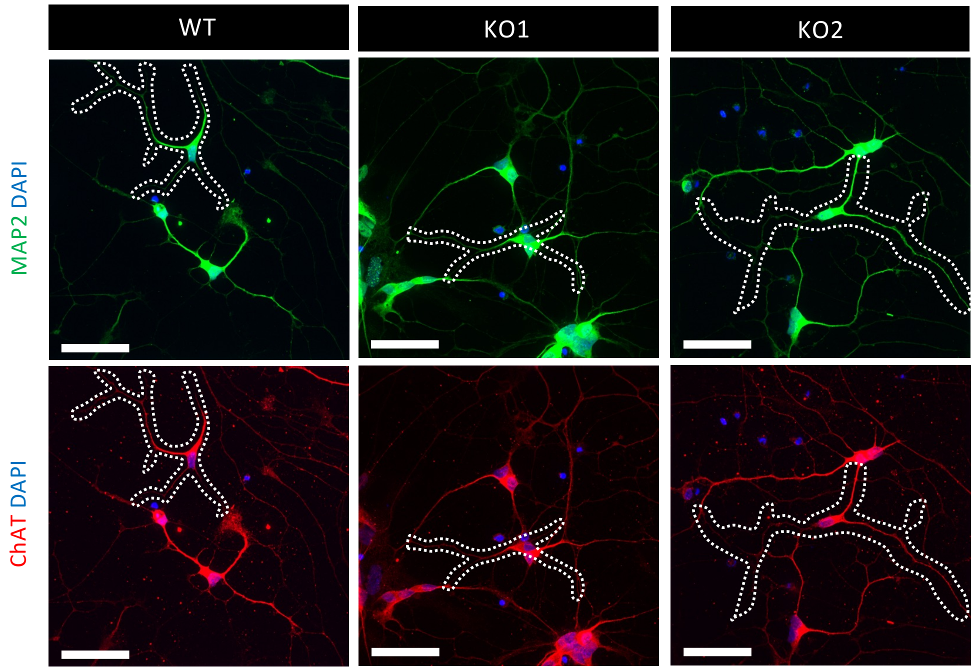

I

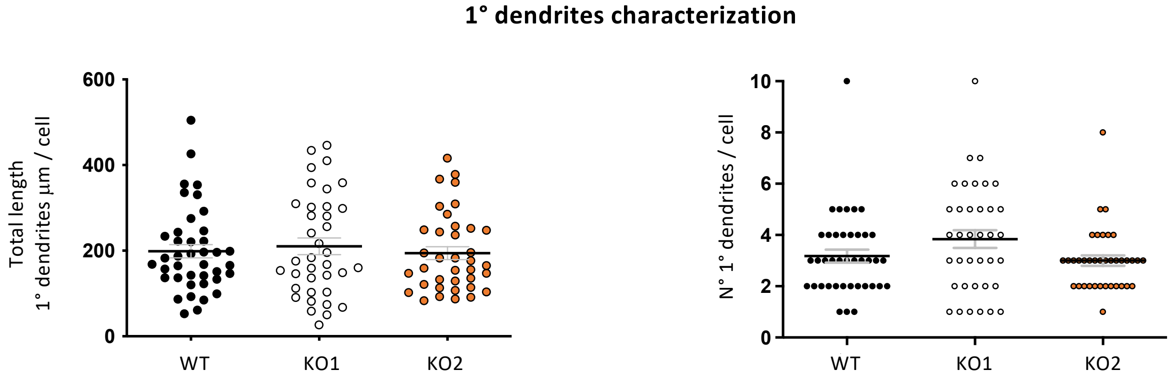

J

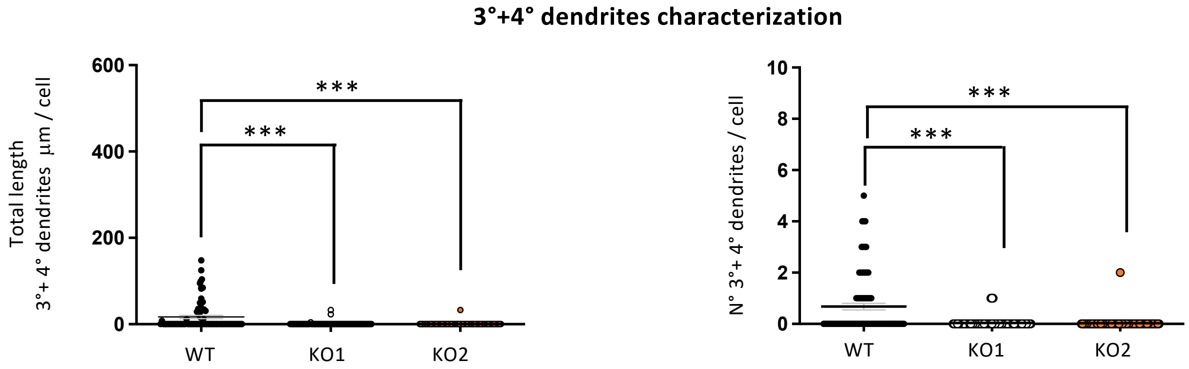

Supplementary Figure 5

A) Left: RT-qPCR quantification of *NCAM1* transcripts in SK-N-BE cells (D 1.5) treated with pcDNA 3.1 $^{(+)}$ empty vector or with pcDNA bearing the coding DNA sequence (CDS) of *NCAM1* mRNA. Data were normalized to GAPDH transcripts and represent means ± SEM of three biological replicates. Middle: representative image of SK-N-BE cells (D 1.5) treated with pcDNA 3.1 $^{(+)}$ empty vector or pcDNA bearing the CDS of *NCAM1* mRNA after 24h of cell scratching. Red boxes highlight the region of interest (ROI) representing the wound area. White lines represent scale bars corresponding to 100 μm. Right: quantification of SK-N-BE cells (D 1.5) treated with pcDNA 3.1 $^{(+)}$ empty vector or with pcDNA bearing the coding DNA sequence (CDS) of *NCAM1* mRNA that migrate in the ROI after 24h of cells scratching and starvation. Each dot represents the counted cells for each acquisition (2 images for each biological replicate). Data represent mean ± SEM of three biological replicates. Paired samples were represented with different symbol shapes. Data information: *$p<0.05$, paired Student's t test.

B) Left: representative image of SK-N-BE cells (D 1.5) treated with LNA-SCR or LNA miR-4492 after 24h of cell scratching. Red boxes highlight the region of interest (ROI) representing the wound area. White lines represent scale bars corresponding to 100 μm. Right: quantification of SK-N-BE cells (D 1.5) treated with LNA-SCR or LNA miR-4492 that migrate in the ROI after 24h of cells scratching and starvation. Each dot represents the counted cells for each acquisition (2 images for each biological replicate). Data represent mean ± SEM of three biological replicates. Paired samples were represented with different symbol shapes. Data information: *$p<0.05$, paired Student's t test.

C) Upper panel: Schematic representation of the donor vector used to block CyCoNP transcription in hiPSCs is shown. HAL and HAR: Left and Right Homology Arms, PAS: Poly Adenylation Signal, Prom: Promoter, NeoR/KanR: Neomycin/Kanamycin resistance cassette. The relative position of the two single guide RNAs (sg1 and 2; sgRNAs) co-transfected to target CyCoNP exon 1 is shown. The two DNA oligos used for hiPSC clones screening are shown (Fw and Rv). See **Materials and Methods** for details. Lower panel: electrophoresis analysis showing the amplification pattern of the CyCoNP locus after transfection and selection of hiPSC colonies. Clones 1, 2 and 3, that display a different pattern of bands compared to the WT, are circled in red.

D) RT-qPCR analysis of *SLC18A3*, *NCAM1* and *CTSV* transcripts in hiPSC-derived MNs at day 8 of differentiation in CyCoNP wild-type (WT) cells and two different CyCoNP knock-out cellular clones (KO1, KO2). Data were normalized to *ATP5O* transcript and represent means ± SEM of three biological replicates.

E) RT-qPCR analysis of miR-4492 expression in hiPSC-derived MN at day 8 of differentiation in CyCoNP wild-type (WT) cells and two different CyCoNP knock-out cellular clones (KO1, KO2). Data were normalized over the U6 snRNA transcript and represent means ± SEM of three biological replicates.

F) Representative confocal captions of immunofluorescence analysis for the dendrites marker MAP2 (green), the neuronal marker Islet 1/2 and DAPI (blue) in CyCoNP WT, KO1 and KO2 hiPSC-derived MN. White lines represent scale bars corresponding to 50 μm.

G) Dot plot representing the mean percentage ± SEM of three biological replicate of Islet positive cells respect to total cell number in CyCoNP WT, KO1 and KO2 hiPSC-derived MN. A range of #766 to #1617 cells were analyzed for each condition.

H) Full field confocal images for cells depicted in **Figure 5D**. Cells were labeled by immunofluorescence for the dendrites marker MAP2 (green), the mature MN marker ChAT (Red) and DAPI (blue). Selected cells analyzed for neurites tracing are marked with dashed lines. White lines represent scale bars corresponding to 50 μm.

I) Left: dot plot representing the values distribution of elongation for sum length of all primary branches (total length of 1° dendrites per cell) in CyCoNP WT, KO1 and KO2 hiPSC-derived MN. Right: dot plot representing the number of 1° dendrites per cell. A total of #40, #37, #37 cells were traced for each condition. Data represent mean ± SEM of three biological replicates.

J) Left: dot plot representing the values distribution of elongation for sum length of all tertiary and quaternary branches (total length of 3°+4° dendrites per cell) in CyCoNP WT, KO1 and KO2 hiPSC-derived MN. Right: dot plot representing the number of 3°+4° dendrites per cell. A total of #40, #37, #37 cells were traced for each condition. Data represent mean ± SEM of three biological replicates.

Data information: ns (non-significant) $p>0.05$, **$p<0.01$, ***$p < 0.001$, unpaired Student's t test.
